# New tools to analyze overlapping coding regions

**DOI:** 10.1186/s12859-016-1389-7

**Published:** 2016-12-13

**Authors:** Amir H. Bayegan, Juan Antonio Garcia-Martin, Peter Clote

**Affiliations:** Biology Department, Boston College, 140 Commonwealth Avenue, Chestnut Hill MA, 02467 USA

**Keywords:** Overlapping coding region, Ribosomal frameshift, Frameshift stimulating signal, HIV-1, HCV

## Abstract

**Background:**

Retroviruses transcribe messenger RNA for the overlapping Gag and Gag-Pol polyproteins, by using a programmed -1 ribosomal frameshift which requires a slippery sequence and an immediate downstream stem-loop secondary structure, together called frameshift stimulating signal (FSS). It follows that the molecular evolution of this genomic region of HIV-1 is highly constrained, since the retroviral genome must contain a slippery sequence (sequence constraint), code appropriate peptides in reading frames 0 and 1 (coding requirements), and form a thermodynamically stable stem-loop secondary structure (structure requirement).

**Results:**

We describe a unique computational tool, RNAsampleCDS, designed to compute the number of RNA sequences that code two (or more) peptides *p*,*q* in overlapping reading frames, that are identical (or have BLOSUM/PAM similarity that exceeds a user-specified value) to the input peptides *p*,*q*. RNAsampleCDS then samples a user-specified number of messenger RNAs that code such peptides; alternatively, RNAsampleCDS can exactly compute the position-specific scoring matrix and codon usage bias for all such RNA sequences. Our software allows the user to stipulate overlapping coding requirements for all 6 possible reading frames simultaneously, even allowing IUPAC constraints on RNA sequences and fixing GC-content.

We generalize the notion of *codon preference index* (CPI) to overlapping reading frames, and use RNAsampleCDS to generate control sequences required in the computation of CPI. Moreover, by applying RNAsampleCDS, we are able to quantify the extent to which the overlapping coding requirement in HIV-1 [resp. HCV] contribute to the formation of the stem-loop [resp. double stem-loop] secondary structure known as the frameshift stimulating signal. Using our software, we confirm that certain experimentally determined deleterious HCV mutations occur in positions for which our software RNAsampleCDS and RNAiFold both indicate a single possible nucleotide. We generalize the notion of codon preference index (CPI) to overlapping coding regions, and use RNAsampleCDS to generate control sequences required in the computation of CPI for the Gag-Pol overlapping coding region of HIV-1. These applications show that RNAsampleCDS constitutes a unique tool in the software arsenal now available to evolutionary biologists.

**Conclusion:**

Source code for the programs and additional data are available at http://bioinformatics.bc.edu/clotelab/RNAsampleCDS/.

**Electronic supplementary material:**

The online version of this article (doi:10.1186/s12859-016-1389-7) contains supplementary material, which is available to authorized users.

## Background

Programmed ribosomal frameshift (PRF) is a curious phenonenon, exploited especially by certain viruses, in order to translate two different protein products from the same messenger RNA. The frameshift is caused by particular sequence and structural elements of the mRNA which sometimes cause the ribosome to slip and readjust the reading frame, thus allowing viruses to pack more information into their genomes. Since the ratio of the protein products coded in overlapping reading frames depends on the PRF efficiency, which has been finely tuned by evolution, any chemical that can modify this efficiency could prove to be a useful anti-viral agent. Though partcularly important for the life cycle of certain viruses, such as HIV-1 and HCV, programmed ribosomal frameshift can be found in all kingdoms of life [1].

**Fig. 1:**
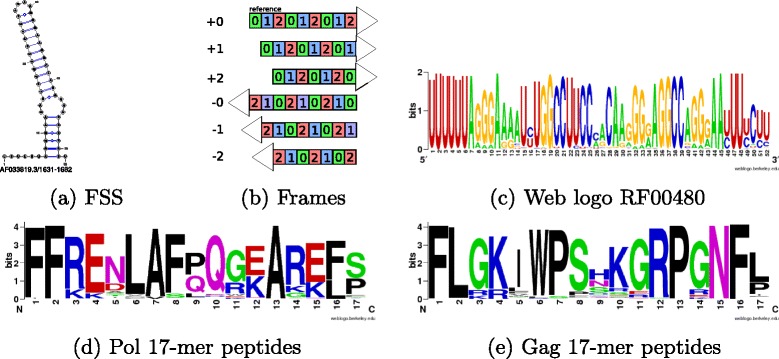
**a** Minimum free energy (MFE) structure of the initial 52-nt Gag-Pol overlapping reading frame in positions 1631-1682 of the HIV-1 complete genome (GenBank AF033819.3). This frameshift stimulating signal (FSS) contains the initial slippery sequence heptamer, given by U UUU UUA in the Gag reading frame, as well as the displayed stem-loop secondary structure, which together promote a programmed -1 frameshift UUU UUU A in the Pol reading frame. **b** Depiction of all 6 possible reading frames – RNAsampleCDS samples RNA sequences that code in all possible reading frames, allowing IUPAC sequence constraints **c** Sequence logo for 145 RNA HIV-1 frameshift signal sequences from the RF00480 seed alignment from Rfam 12.0 [4]. **d** Sequence logo for the Pol peptide coded by 138 RNA HIV-1 frameshift signal sequences from the RF00480 seed alignment from Rfam 12.0; Pol peptide translated from nucleotide positions 1-51. **e** Sequence logo for the Gag peptide coded by 138 RNA HIV-1 frameshift signal sequences from the RF00480 seed alignment from Rfam 12.0; Gag peptide translated from nucleotide positions 2-52. Since some sequences from RF00480 contained IUPAC codes for uncertain data, the data were disambiguated–for instance, the code B (not A) was disambiguated by randomly assigning either C,G or U with probability 1/3. Seven sequences were removed from the seed alignment of 145 RNAs due to gaps in the alignment, and another five sequences were removed since either the Pol or Gag peptide contained a stop codon–resulting in 133 sequences for nucleotide analysis. Peptide sequence logos for the 138 Pol and Gag peptides were created using WebLogo [26]

In HIV-1, Pol is obtained from a fused Gag-Pol polyprotein via a programmed -1 ribosomal frameshift, which naturally occurs with a frequency of 5–10%; moreover, an increase of ribosomal frameshift frequency is associated with a decrease in viral infectivity [2]. The -1 ribosomal frameshift is caused by two *cis*-acting RNA elements, together known as *frameshift stimulating signal* (FSS): (1) a heptameric *slippery sequence* (U UUU UUA), where the Gag reading frame is indicated, and (2) a downstream stem-loop secondary structure, often with either internal loop or right bulge. The FSS from HIV-1 genome (AF033819.3/1631-1682) is shown in Fig. 1 a, where the minimum free energy (MFE) secondary structure was determined by RNAfold from *Vienna RNA Package* 2.1.9 [3]. The Pol reading frame is -1 with respect to the Gag reading frame, or equivalently, the Gag reading frame is +1 with respect to the Pol reading frame (convention adopted throughout this paper) – Fig. 1 b depicts the six reading frames considered in this paper. While the entire Gag-Pol overlap region in HIV-1 AF033819.3 is from position 1631 to 1838 (Pr55 Gag polyprotein is coded at AF033819.3/336-1838), the 17-mer Pol [resp. Gag] peptide coded in the 52 nt FSS region 1631-1682 is FFREDLAFLQGKAREFS [resp. FLGKIWPSYKGRPGNFL]. Moreover, we found the secondary structure from Fig. 1a to be the most common MFE structure for 52 nt segments of the Pol coding region, which begin by UUUUUUA, taken from the HIV Sequence Database in Los Alamos National Laboratory (LANL) available at www.hiv.lanl.gov. Due to its importance, a collection of 145 HIV-1 ribosomal frameshift elements is given in the family RF00480 in Rfam 12.0 [4]. Figure 1 c displays the sequence logo obtained from the 145 sequences in the seed alignment of RF00480, while Fig. 1 d and e respectively display the sequence logos for the 17-mer Pol and Gag peptides coded in RF00480.

For decades, research in evolutionary biology has focused mostly on protein-coding regions, leading to the development of sophisticated computational tools, such as PAML [5] and HYPHY [6], to compute the ratio *d* *N*/*d* *S* of non-synonomous mutation rate *dN* to the synonomous mutation rate *dS* [7–9]. Pedersen and Jenson [10] extended the codon substitution model of Goldman and Yang [8] to overlapping genes in a site-specific manner, where evolutionary constraints of both genes are taken into account. However, estimation of evolutionary parameters in this model required computationally expensive Markov chain Monte Carlo simulations. By dropping the condition of site specificity, Sabath et al. [11] were able to apply a maximum likelihood method to estimate parameters in a more efficient manner. The resulting tool has been used to predict functionality of overlapping reading frames [12]. An evolutionary model has been developed for coding regions with conserved RNA secondary structures [13] as well. This approach was used to determine the effects of structural elements on nucleotide substitution in hepatitis C virus.

Several methods have been developed to sample sequences using an evolutionary model derived from a given phylogeny [14–16]. To the best of our knowledge, however, there is no previously published method for sampling sequences in overlapping coding regions. The program SISSI [16] incorporates a user-defined system of dependencies between the nucleotides; however, it is not possible using SISSI to sample sequences that code in overlapping reading frames, since SISSI requires that any position in an RNA sequence must belong to a single codon. Moreover, SISSI does not allow sequence and structural dependencies to be specified simultaneously. Our work in this paper is orthogonal to the foregoing computational models and tools of mathematical evolution theory and does not rely on phylogeny information. In full generality, the new software RNAsampleCDS supports the following. For each reading frame *r*∈{+0,+1,+2,−0,−1,−2} illustrated in Fig. 1 b, let *p*_*r*_ be a length *n* sequence in the 22-letter alphabet consisting of IUPAC codes for each amino acid, together with symbol *X* (any residue) and *O* (any residue or STOP). RNAsampleCDS computes the number of RNA sequences *a*_0_,…,*a*_3*n*+2_ which simultaneously code protein $p^{\prime }_{r}$ in reading frame *r*, such that either $p^{\prime }_{r}$ is identical to *p*_*r*_, or (optionally) whose BLOSUM/PAM similarity to *p*_*r*_ exceeds a user-specified value. (Throughout the article, we say that the peptide *p* is *BLOSUM[PAM] θ similar* to another peptide *p* ^′^, if each amino acid of *p* has BLOSUM[PAM resp.] similarity of *at least* *θ* with the corresponding amino acid of *p* ^′^.) RNAsampleCDS can then compute the PSSM and codon usage frequency for such proteins, as well as sample a user-specified number of such sequences. RNAsampleCDS runs in linear time and space, although if GC-content is optionally controlled, then time and space requirements are quadratic. For expository reasons, we describe the algorithms for only two proteins *p*,*q* respectively in reading frame 0 and 1; however, our code is general as just described – see the Additional file 1 for details on the general algorithm. Using RNAsampleCDS, we undertake a preliminary analysis of the Gag-Pol overlapping reading frame in human immunodeficiency virus (HIV-1) and of the triple overlapping reading frame of hepatitis C virus (HCV).

## Methods

### RNAsampleCDS

Let *p*=*p*_1_,…,*p*_*n*_ and *q*=*q*_1_,…,*q*_*n*_ be two peptides of equal length. In this section, we are interested in the following questions.

1. Which sequences *a*_0_,…,*a*_3*n*_ of messenger RNA translate the peptide *p* in reading frame 0 and also translate the peptide *q* in reading frame +1?
2. Which sequences *a*_0_,…,*a*_3*n*_ of messenger RNA translate peptides *p* ^′^=*p*1′,…,*p* *n*′ in reading frame 0 and peptide *q* ^′^=*q*1′,…,*q* *n*′ in reading frame +1, where the BLOSUM/PAM similarity of *p* with *p* ^′^ and *q* with *q* ^′^ is greater than or equal to a user-specified threshold *θ*?
3. What is the profile, or PSSM, for the collection of mRNAs from (1) and (2)?
4. What is the total number of sequences satisfying (1) and (2), and how can we sample sequences *a*_0_,…,*a*_3*n*_ of messenger RNA in an unbiased manner, in order to satisfy either (1) or (2)?

By developing software to sample mRNA sequences that code user-specified proteins in different reading frames, we can then analyze the samples with other tools to provide an estimate of the probability of satisfying a given property of interest, hence give approximate answers for questions like the following: What is the expected stem size in the minimum free energy (MFE) structure of RNAs that translate peptides *p* ^′^,*q* ^′^ in reading frames 0,1, where the BLOSUM/PAM similarity of *p*,*p* ^′^ and of *q*,*q* ^′^ is at least a user-specified threshold value of *θ*? As we show, it is not difficult to see that questions (1,2) are easily answered using breadth first search (BFS); however, for large values of *n*, it can happen that BFS in not practical, since the number of messenger RNAs can be of size exponential in *n*. For that reason, we describe a novel dynamic programming (DP) algorithm to answer questions (3) and (4).

We first need a few definitions. If *xyz* is a trinucleotide, then let *t* *r*(*x* *y* *z*) denote the amino acid whose codon is *xyz* in the genetic code; i.e. *t* *r*(*x* *y* *z*) is the amino acid translated from codon *xyz*, unless *xyz* is a stop codon. If *xyzu* is a tetranucleotide, then let *t* *r*_0_(*x* *y* *z* *u*) [resp. *t* *r*_1_(*x* *y* *z* *u*)] denote the amino acid whose codon is *xyz* [resp. *yzu*]; i.e. *t* *r*_0_(*x* *y* *z* *u*)=*t* *r*(*x* *y* *z*) and *t* *r*_1_(*x* *y* *z* *u*)=*t* *r*(*y* *z* *u*). For each *k*=1,…,*n*, define the collection *L*_*k*_ of 4-tuples *s*=*s*_0_,*s*_1_,*s*_2_,*s*_3_ such that *t* *r*_0_(*s*)=*t* *r*(*s*_0_,*s*_1_,*s*_2_)=*p*_*k*_ and *t* *r*_1_(*s*)=*t* *r*(*s*_1_,*s*_2_,*s*_3_)=*q*_*k*_. Define two 4-tuples *s*=*s*_0_ *s*_1_ *s*_2_ *s*_3_ and *t*=*t*_0_ *t*_1_ *t*_2_ *t*_3_ to be *compatible* if *s*_3_=*t*_0_ – i.e. the tail of *s* equals the head of *t*. Note that if 4-tuples *s*,*t* are compatible, then the *merge* *s*_0_,*s*_1_,*s*_2_,*t*_0_,*t*_1_,*t*_2_,*t*_3_ of *s*,*t* has the property that amino acids are translated by each of the four codons *s*_0_ *s*_1_ *s*_2_, *s*_1_ *s*_2_ *s*_3_, *t*_0_ *t*_1_ *t*_2_, and *t*_1_ *t*_2_ *t*_3_.



Using our implementation of the BFS approach in Algorithm 1, we can easily determine that there are exactly 32 52-nt RNAs that translate the 17-residue Pol peptide FFREDLAFLQGKAREFS in reading frame 0, and the 17-residue Gag peptide FLGKIWPSYKGRPGNFL in reading frame +1. These 17-mer peptides are those which constitute the beginning of the Gag-Pol overlap in the HIV-1 genome (nucleotides 1631-1682 in GenBank AF033819.3). The entire Gag-Pol overlap region is from 1631-1835, whereby the 68-mer Pol [resp. Gag] peptide is coded in the region 1631-1834 [resp. 1632-1835 with a Gag STOP codon at 1836-1838]. Our implementation of the BFS method returns exactly 256 205-nt RNAs that code the Pol [resp. Gag] 68-mers from HIV-1 (GenBank AF033819.3).

**Fig. 2:**
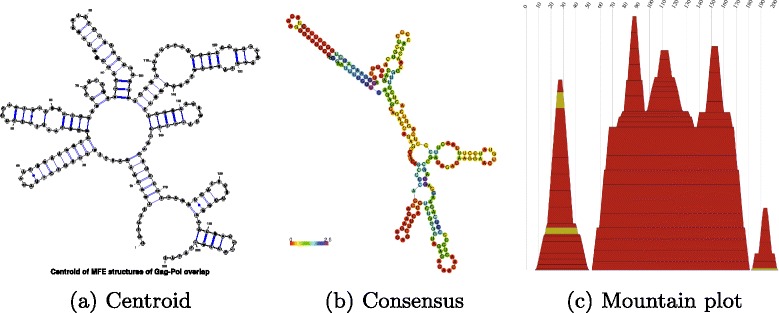
**a** The centroid secondary structure, **b** RNAalifold consensus structure, and **c** the corresponding mountain plot for the alignment of all 256 205-nt RNA sequences that code the Pol and Gag 68-mer peptides from HIV-1 (Pol 1631-1835, Gag 1632-1836 in GenBank AF033819.3)

Figure 2 displays the centroid secondary structure, RNAalifold [17] consensus structure, and the corresponding mountain plot for the alignment of all 256 205-nt RNA sequences that code the Pol and Gag 68-mer peptides from HIV-1 (Pol 1631-1835, Gag 1632-1836 in GenBank AF033819.3), *not* necessarily containing the slippery sequence UUUUUUA.

Further analysis (data not shown) indicates that there is considerable variation in the low energy structures of RNAs that exactly code the same 68-mer Pol and Gag peptides as those coded by AF033819.3/1631-1836.

Question (2) is an obvious generalization of (1), and is easy to answer by generalizing the collection *L*_*k*_ of 4-tuples *s*=*s*_0_,*s*_1_,*s*_2_,*s*_3_ such that *t* *r*_0_(*s*)=*t* *r*(*s*_0_,*s*_1_,*s*_2_)=*p* *k*′ and *t* *r*_1_(*s*)=*t* *r*(*s*_1_,*s*_2_,*s*_3_)=*q* *k*′, where the BLOSUM/PAM similarity of *p*_*k*_,*p* *k*′ and of *q*_*k*_,*q* *k*′ is at least a user-specified threshold *θ*.

It is more interesting to turn to question (3), which requires a different strategy, since the number of RNAs returned by BFS may be exponentially large. Indeed, if RNA sequences are required to code peptides *p* [resp. *q*] whose amino acids have BLOSUM62 similarity of at least *θ* to those of the Pol [resp. Gag] 17-mer peptide coded in reading frame 0 [resp. 1] in AF033819.3/1631-1682, then the number of solution sequences is 256 (*θ*=4), 34,560 (*θ*=3), 90,596,966,400 (*θ*=2), 2.14285987145e+32 (*θ*=1), 3.61150917928e+56 (*θ*=0), 1.20555937201e+81 (*θ*=−1), 1.17643153215e+106 (*θ*=−2)! To address question (3), define the forward and backwards partition function *ZF*, *ZB* as follows.

- **Forward partition function:** For integer *k*=1,…,*n* and nucleotide *c* *h*∈{*A*,*C*,*G*,*U*}, define *Z* *F*(*k*,*c* *h*) to be the number of RNAs **a**=*a*_0_,…,*a*_3*k*_ such that *a*_3*k*_ is the nucleotide *ch*, and **a** translates the peptide *p*_1_,…,*p*_*k*_ resp. *q*_1_,…,*q*_*k*_ in reading frame 0 resp. 1; i.e. *t* *r*_0_(**a**)=*p*_1_,…,*p*_*k*_ and *t* *r*_1_(**a**)=*q*_1_,…,*q*_*k*_.
- **Backward partition function:** For integer *k*=1,…,*n* and nucleotide *c* *h*∈{*A*,*C*,*G*,*U*}, define *Z* *B*(*k*,*c* *h*) to be the number of RNAs **a**=*a*_3*k*_,*a*_3*k*+1_,…,*a*_3*n*_ such that *a*_3*k*_ is the nucleotide *ch*, and **a** translates the peptide *p*_*k*_,…,*p*_*n*_ resp. *q*_*k*_,…,*q*_*n*_ in reading frame 0 resp. 1; i.e. *t* *r*_0_(**a**)=*p*_*k*_,…,*p*_*n*_ and *t* *r*_1_(**a**)=*q*_*k*_,…,*q*_*n*_.

By dynamic programming, it is straightforward to compute the forward and backward partition functions in linear time and space, as done in Algorithm 2.

Recall that the *indicator function* *I*[boolean condition] returns the value 1 if the boolean condition within its scope is true, and otherwise the value returned is 0.

By appropriately redefining *L*_*k*_, the recursions of Algorithm 2 can easily be modified to instead count the number of sequences coding $p^{\prime }_{1},\ldots,p'_{n}$ in reading frame 0 and $q^{\prime }_{1},\ldots,q'_{n}$ in reading frame +1, such that for each *i*, the BLOSUM/PAM similarity of *p*_*i*_,*p* *i*′ and of *q*_*i*_,*q* *i*′ exceeds a user-specified threshold *θ*, or for which the Kyte-Doolittle hydrobicity of *p*_*i*_,*p* *i*′ and *q*_*i*_,*q* *i*′ differ by at most a user-specified upper bound, etc. The same remark applies to *all* algorithms of this section, although for reasons of space, we do not explicitly mention such extensions. Nevertheless, such extensions are supported by the software RNAsampleCDS.

By refining the definition of forward and backward partition function, Algorithms 1 and 2 can be modified to keep track of the GC-content, albeit at an overhead for the space required. For an arbitrary RNA sequence **a**, let *g* *c* *c* *o* *u* *n* *t*(**a**) denote the number of Gs or Cs occurring in **a**.

- **Forward partition function accounting for GC-content:** For integer *k*=1,…,*n* and nucleotide *c* *h*∈{*A*,*C*,*G*,*U*}, define *Z* *F*_*GC*_(*k*,*x*,*c* *h*) to be the number of RNAs **a**=*a*_0_,…,*a*_3*k*_ such that *a*_3*k*_ is the nucleotide *ch*, *g* *c* *c* *o* *u* *n* *t*(**a**)=*x*, and **a** translates the peptide *p*_1_,…,*p*_*k*_ resp. *q*_1_,…,*q*_*k*_ in reading frame 0 resp. 1; i.e. *t* *r*_0_(**a**)=*p*_1_,…,*p*_*k*_ and *t* *r*_1_(**a**)=*q*_1_,…,*q*_*k*_.
- **Backward partition function accounting for GC-content:** For integer *k*=1,…,*n* and nucleotide *c* *h*∈{*A*,*C*,*G*,*U*}, define *Z* *B*_*GC*_(*k*,*x*,*c* *h*) to be the number of RNAs **a**=*a*_3*k*_,*a*_3*k*+1_,…,*a*_3*n*_ such that *a*_3*k*_ is the nucleotide *ch*, *g* *c* *c* *o* *u* *n* *t*(**a**)=*x*, and **a** translates the peptide *p*_*k*_,…,*p*_*n*_ resp. *q*_*k*_,…,*q*_*n*_ in reading frame 0 resp. 1; i.e. *t* *r*_0_(**a**)=*p*_*k*_,…,*p*_*n*_ and *t* *r*_1_(**a**)=*q*_*k*_,…,*q*_*n*_.

Though not explicitly described, *all* the following algorithms (PSSM computation and sampling) can be modified to account for GC-content. Our program, RNAsampleCDS, implements all the algorithms described in this section, including versions that account for GC-content. Moreover, our program supports any *two or more* overlapping coding regions in any of the 6 reading frames – i.e. reading frame 0,1,2 on the plus-strand and 0,1,2 on the minus-strand, as shown in Fig. 1 b.

Note that an easy modification of the above algorithm allows one to compute the total number of RNAs of length 3*n*+1, which code *n*-mer peptides *p* [resp. *q*] in reading frames 0 [resp. 1], i.e. for which neither reading frame contains a stop codon. This modification is later used to compute the probability that a random RNA of length 3*n*+1 will code in both reading frames 0 and 1. Algorithm 3 applies Algorithm 2 in order to compute the exact value of the position specific scoring matrix (PSSM).



The recursions can be easily modified, if the RNA sequence is instead required to code $p^{\prime }_{1},\ldots,p'_{n}$ in reading frame 0 and $q^{\prime }_{1},\ldots,q'_{n}$ in reading frame +1, such that for each *i*, the BLOSUM/PAM similarity of *p*_*i*_,*p* *i*′ and of *q*_*i*_,*q* *i*′ exceeds a user-specified threshold *θ*. This answers question (3). The resulting DP program is very fast, since the run time is linear in *n*, while the BFS program has run time that is exponential in *n*.

Given a gapless alignment *S* of mRNA sequences of length 3*n*+1, each of which codes a protein in reading frame 0 and 1, define the *positional codon frequency* *P* *C* *F*(*w*,*k*,*r*) to be the number of occurrences of *w* in the *k*th codon position in reading frame *r*∈{0,1} of a sequence in *S*. If *S* is the collection of all mRNAs that code proteins *p*,*q* respectively in reading frame 0,1, which are identical to (or alternatively have BLOSUM/PAM similarity that exceeds threshold *θ*), then the positional codon frequency can be defined from the partition functions *Z* *F*,*Z* *B* as done in Algorithm 4.



Next, in order to sample RNA sequences that code peptides *p*=*p*_1_,…,*p*_*n*_ resp. *q*=*q*_1_,…,*q*_*n*_ in reading frames 0 resp. 1, we construct the sampled sequence from last to first character, each time ensuring that *Z* *F*(*k*,*c* *h*)>0 where *ch* is the leading character of the current sample *a*_3*k*−1_,*a*_3*k*_,…,*a*_3*n*_. This is described in done in Algorithm 5, where we recall that *L*_*k*_ denotes the collection of 4-tuples *s*=*s*_0_,*s*_1_,*s*_2_,*s*_3_ such that *t* *r*_0_(*s*)=*t* *r*(*s*_0_,*s*_1_,*s*_2_)=*p* *k*′ and *t* *r*_1_(*s*)=*t* *r*(*s*_1_,*s*_2_,*s*_3_)=*q* *k*′, and the BLOSUM/PAM similarity of *p*_*k*_,*p* *k*′ and of *q*_*k*_,*q* *k*′ is at least a user-specified threshold *θ*.



It is straightforward to modify the previous algorithm to sample in a *weighted* fashion as done in Algorithm 6. First, recall that *L*_*k*_ denotes the collection of 4-tuples *s*=*s*_0_,*s*_1_,*s*_2_,*s*_3_ such that *t* *r*_0_(*s*)=*t* *r*(*s*_0_,*s*_1_,*s*_2_)=*p* *k*′ and *t* *r*_1_(*s*)=*t* *r*(*s*_1_,*s*_2_,*s*_3_)=*q* *k*′, and the BLOSUM/PAM similarity of *p*_*k*_,*p* *k*′ and of *q*_*k*_,*q* *k*′ is at least a user-specified threshold *θ*. Additionally, if *c* *h*∈{*A*,*C*,*G*,*U*} then let *L*_*k*,*ch*_ denote the set of tuples *t* in *L*_*k*_, whose last element *t*_3_ is *ch*.



Our implementation of the algorithms described in this section allows the user to stipulate *sequence constraints* using any IUPAC nucleotide codes, for instance, designating the first 7 nucleotides to be the slippery sequence UUUUUUA, or to consist of an alternation of purines and pyrimidines RYRYRYR, etc.

Finally, we note that all the previous algorithms in this section can be extended to handle *multiple* overlapping reading frames in all six reading frames, i.e. reading frames +0,+1,+2 on the plus strand and reading frames -0,-1,-2 on the minus strand, as illustrated in Fig. 1 b. For instance, in order to compute the forward partition function for reading frames 0,1,2 we define *Z* *F*(*k*,*c* *h*1,*c* *h*2) to be the number of RNA sequences **a** of length 3*k*+2 whose last two nucleotides are *c* *h*1,*c* *h*2, such that *t* *r*_0_(**a**)=*p*_1_,…,*p*_*k*_, *t* *r*_1_(**a**)=*q*_1_,…,*q*_*k*_, *t* *r*_2_(**a**)=*r*_1_,…,*r*_*k*_, for user-specified peptides **p**=*p*_1_,…,*p*_*n*_, **q**=*q*_1_,…,*q*_*n*_, **r**=*r*_1_,…,*r*_*n*_. Now we define *L*_*k*_ to be the set of 5-tuples *s*=*s*_0_,…,*s*_4_ such that *s*_0_ *s*_1_ *s*_2_ codes residue *p*_*k*_, *s*_1_ *s*_2_ *s*_3_ codes residue *q*_*k*_, and *s*_2_ *s*_3_ *s*_4_ codes residue *r*_*k*_. The definition of the generalization of the forward partition function *Z* *F*(*k*,*c* *h*1,*c* *h*2), analogous to that defined in Algorithm 2, is as follows:

- Case 1: *k*=1. Then *Z* *F*(*k*,*c* *h*1,*c* *h*2) equals
$\sum \limits _{s_{0}s_{1}s_{2}s_{3}s_{4} \in L_{k}} I[ s_{3}=ch1,s_{4}=ch2 ]$
- Case 2: *k*=2,…,*n*2,…,*n*. Then *Z* *F*(*k*,*c* *h*1,*c* *h*2) equals
$\sum \limits _{s_{0}s_{1}s_{2}s_{3}s_{4} \in L_{k}} I[ s_{3}=ch1,s_{4}=ch2 ] \cdot ZF(k-1,s_{0},s_{1})$

Our publicly available code RNAsampleCDS supports all the above described variants of Algorithms 1-6 with possible IUPAC sequence constraints, stipulation of GC-content, and where the user may stipulate that particular peptides are coded in any or all of the six reading frames displayed in Fig. 1 b. See Additional file 1 for details of how we determine the run time estimate of ≈0.58831373·*L*+0.00550239·*N* to generate compute the partition function and generate *N* samples of RNA sequences of length *L* that code any peptide in each of the six possible reading frames.

## Results and Discussion

In this section, we use RNAsampleCDS to study novel aspects of human immunideficiency virus HIV-1 and hepatitis C virus HCV, that cannot be determined using methods other than those described in this paper.

### HIV-1 programmed -1 frameshift

**Fig. 3:**
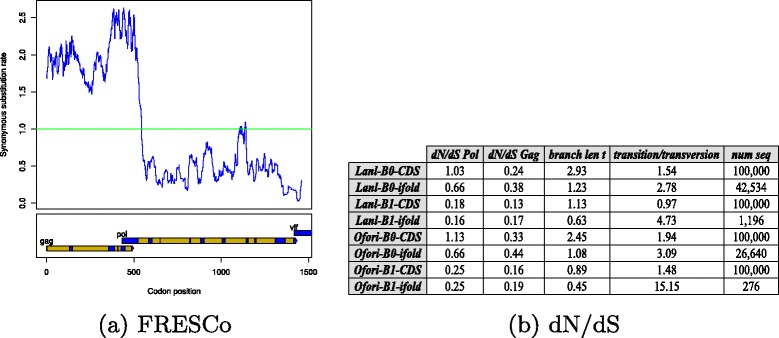
**a** Output from the program FRESCo [18], when run on the Gag reading frame of an alignment of 200 sequences from the LANL HIV-1 database using 50 nt windows. Note the precipitous drop in dN/dS value at the beginning of Gag-Pol overlap region. **b** Values of *d* *N*/*d* *S*, branch length, and transition/transversion rate (see [8] for definitions) for the 52 nt Gag-Pol overlap regions within a multiple alignment from Rfam family RF00480 as well as from 52 nt RNA sequences generated by the programs RNAsampleCDS and RNAiFold. These programs generate sequences that code peptides, each of whose amino acids has BLOSUM62 similarity of either 0 or 1 to the corresponding amino acids in the Gag [resp. Pol] reading frame of the peptide translated by the 52 nt HIV-1 overlap region of [2] or by GenBank accession code AF033819.3/1631-1681. The program RNAsampleCDS ensures only coding requirements, while RNAiFold ensures both coding requirements and that the 52 nt RNAs fold into the minimum free energy structure of the Gag-Pol overlap region of HIV-1 from [2] and GenBank accession code AF033819.3/1631-1682

Analysis of HIV-1 overlap: Since HIV-1 and other retroviruses have a -1 ribosomal frameshift in the initial portion of the Gag-Pol overlap, this can be detected by the software FRESCo [18], which predicts regions of excess synonymous constraint in short, deep alignments. Figure 3 a displays the dN/dS ratio we obtained for HIV-1 AF033819.3 with respect to the Gag reading frame, when aligned with other HIV-1 genomes from the Los Alamos HIV Database – see also Additional file 1: Figure S1. This figure indicates that there is *positive selection* in the Gag region before the Gag-Pol overlap. In contrast, starting with the beginning of the Gag-Pol overlap (nucleotide 1631), there is *purifying selection*; i.e. Fig. 3 a suggests the presence of an important signal starting around position 1631. Figure 3 b displays the *d* *N*/*d* *S* ratio of the 52 nt Gag-Pol overlap region, for both the Gag and Pol reading frames, using the method of [11] which computes a rate matrix for overlapping reading frames – an aspect ignored by PAML and other software. Since Sabath’s program computes *d* *N*/*d* *S* from a pairwise alignment, which is wholly inappropriate for the short 52 nt sequences considered here, we modified the approach by first producing multiple alignments of 52 nt Gag-Pol overlap regions, and then computed the number of (observed) synonomous and nonsynonomous mutations within the Gag [resp. Pol] reading frame, taking account for all codon pairs in the same column. We then modified Sabath’s Matlab program to compute *d* *N*/*d* *S* by maximum likelihood using counts obtained from the multiple alignments. The multiple alignments considered in Fig. 3 b are from Rfam family RF00480 and from 52 nt RNA sequences generated by the programs RNAsampleCDS and RNAiFold 2.0. RNAsampleCDS generates 52 nt sequences, that translate peptides in the Gag [resp. Pol] reading frame, each of whose amino acids has BLOSUM62 similarity of either 0 or 1 to the corresponding amino acids in the Gag [resp. Pol] reading frame of the peptides translated by the 52 nt HIV-1 overlap region of AF033819.3/1631-1682. RNAiFold 2.0 generates 52 nt sequences, that not only satisfy the same coding requirements as RNAsampleCDS, but which also fold into the minimum free energy secondary structure shown in Fig. 1 a. In each case, RNAiFold 2.0 generates *all* sequences that satisfy both the coding and structure requirements, their number being substantially less than the 100,000 sequences generated by RNAsampleCDS. Note the presence of purifying selection for the Gag reading frame, as indicated by *d* *N*/*d* *S* values less than 1.

***Codon preference index:*** In this section, we generalize the notion of *codon preference index* (CPI) [19] to the context of overlapping coding regions. For RNA sequence **a**=*a*_0_,…,*a*_3*n*_ which codes n-mer peptides in reading frames 0, 1, for codon *w*∈({*A*,*C*,*G*,*U*})^3^ and reading frame *r*∈{0,1}, define *f*_(*w*,**a**,*r*)_ to be the number of occurrences of codon *w* in reading frame *r* of **a**, and for amino acid *AA*, define *f*_(*AA*,**a**,*r*)_ to be the number of occurrences of codons coding *AA* in reading frame *r* of **a**. Define the *observed codon preference* in **a** by $p_{obs}(w,\textbf {a}) = \sum _{r=0}^{1} f_{(w,\textbf {a},r)}/\sum _{r=0}^{1} f_{(AA,\textbf {a},r)}$. If *S*is a set of mRNAs of length 3*n*+1, each of which codes *n*-mer peptides in both reading frames 0,1, then define the *observed codon preference* in *S* by $p_{obs}(w,S) = \sum _{r=0}^{1}\sum _{\textbf {a} \in S} f_{(w,\textbf {a},r)}/ \sum _{r=0}^{1} \sum _{\textbf {a} \in S} f_{(AA,\textbf {a},r)}$. Note that *p*_*obs*_(*w*,*S*) is the *probability* that codon *w* will be used for amino acid *AA* in the collection *S* of overlapping coding sequences. Finally, define the *codon preference index* *I*(*w*) of codon *w* in *S* by *I*(*w*)=*p*_*obs*_(*w*,*S*)/*p*_*obs*_(*w*,*S* ^′^), where *S* ^′^ is a *control* set of mRNAs of length 3*n*+1.

**Fig. 4:**
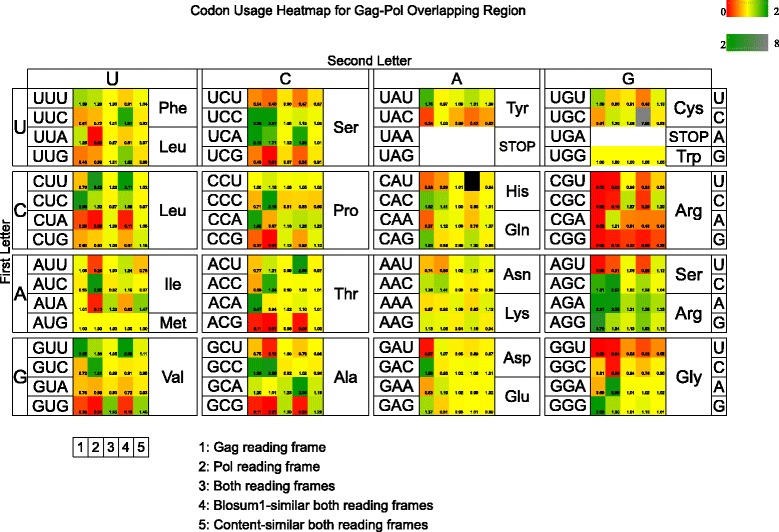
Heat map of the *codon preference index* (CPI) for a collection of 5125 entire Gag-Pol overlap regions of average length 205±10 extracted from LANL HIV-1 database. CPI values shown at bottom right of each square. See text for additional explanation

With these notations, Fig. 4 depicts a heat map for the codon preference index *I*(*w*), computed over 5,125 entire Gag-Pol overlap regions of average length 205±10 (Gag and Pol peptide size ≈68) extracted from LANL HIV-1 database, each starting with the slippery sequence UUUUUUA and terminating with the last Gag codon; additionally the heat map includes Gag-only and Pol-only values for the same overlap region. For this figure, the control set *S* ^′^ is defined differently for each column 1−5, although in all cases, each sequence in *S* ^′^ contains the initial slippery sequence UUUUUUA. For column 1 [resp. 2] *S* ^′^ is the set of all mRNAs that code proteins in the Gag [resp. Pol] reading frame that are coded by some sequence of *S*. For column 3, *S* ^′^ is the set of all mRNAs that code proteins *p* and *q* that are identical to proteins coded in the Gag and Pol reading frames of some sequence **a** of *S*. For column 4, *S* ^′^ is defined as in the case for column 3, except that ‘identical to’ is replaced by ‘BLOSUM62 +1 similar to’. For column 5, *S* ^′^ is the set of all mRNAs that code proteins *p* and *q* that are BLOSUM62 +1 similar to proteins coded in the Gag and Pol reading frames of a sequence **a** of *S*, and whose GC-content lies in the range of GC-content of **a**±5. The heat map of Fig. 4 shows that for serine, *I*(*A* *G* *U*,*G* *a* *g*)<*I*(*A* *G* *U*,*P* *o* *l*)<*I*(*A* *G* *U*,*G* *a* *g*/*P* *o* *l*)≈1; for valine, *I*(*G* *U* *G*,*G* *a* *g*)<1<*I*(*G* *U* *U*,*G* *a* *g*) but *I*(*G* *U* *G*,*G* *a* *g*/*P* *o* *l*)>1>*I*(*G* *U* *U*,*G* *a* *g*/*P* *o* *l*); for proline, *I*(*C* *A* *U*,*G* *a* *g*)<*I*(*C* *A* *U*,*P* *o* *l*)<*I*(*C* *A* *U*,*G* *a* *g*/*P* *o* *l*)≈1, but when the control set is taken to be BLOSUM62 +1 similar peptides to Gag and Pol, then *I*(*C* *A* *U*,*G* *a* *g*/*P* *o* *l*+1)≫1. See Additional file 1: Figures S2 and S3 and the text from Additional file 1 for more detailed explanation. These figures show that the codon usage bias observed at the Gag-Pol junction is not due to natural selection [20] or to the underlying mutational bias, but rather imposed by the overlapping coding constraints.

***Overlapping coding and stem-loop formation:*** Here we describe how to quantify the extent to which coding HIV-1 17-mer peptides in overlapping reading frames induces a stem-loop structure. In particular, we consider the following questions.

1. What is the probability that random RNA forms a stem-loop structure?
2. What is the probability that RNA forms a stem-loop structure, if it is required to code (any arbitrary) peptides in reading frames 0 and 1?
3. What is the probability that RNA forms a stem-loop structure, if it is required to code peptides in reading frames 0 and 1, which are *similar* to peptides coded in the HIV-1 frameshift stimulating signal (FSS)?
4. To what extent do HIV-1 coding requirements in the Pol-Gag overlap region alone induce stem-loop formation?
5. What is the (conditional) probability of coding peptides in reading frames 0 and 1 if the RNA forms a secondary structure similar to the FSS stem-loop structure of HIV-1?

To answer question 1, we generated 200,000 52-nt RNAs, where the first seven nucleotides constituted the slippery sequence UUUUUUA, and each nucleotide in position 8 through 52 was randomly selected with probability 0.25 for each of A,C,G,U. Using RNAshapes, cf. [21], we determined the Boltzmann probability that each RNA sequence has shape **[ ]** [22], i.e. $P(\,\textbf {\texttt {[ ]}}\,) = \sum _{s} \exp (-E(s)/RT)$, where the sum is taken over all *stem-loop* secondary structures, which may contain internal loops and bulges, but no multiloops or multiple stem-loops. Throughout the sequel of the paper, the probability that a given RNA sequence will form a *stem-loop* structure is identified with *P*(**[ ]**). A finer analysis could consider type 1 shapes of the form _ **[** _ **[ ]** _ **]** or _ **[** **[ ]** _ **]**, corresponding to a stem loop with internal loop or right bulge, with left flanking unpaired region, but in this paper we consider only the type 5 stem loop shape [ ]. By *MFE stem-loop structure*, we mean the stem-loop secondary structure which has the minimum free energy, taken over all stem-loop structures. Similarly, *stem-loop MFE* means the minimum free energy of all stem-loop structures. Note that the stem-loop MFE is not necessarily equal to the MFE, since it is possible that a structure having two or more external loops, or containing a multiloop, could have lower energy than that of any stem-loop structure. By uniformly sampling 200,000 52 nt RNAs with no coding requirements, we estimate an average probability of stem-loop formation of 60.7*%* with standard deviation of 36.2*%*, and average stem-loop MFE was −7.65 kcal/mol with standard deviation 3.42 kcal/mol – again, this is for 52 nt RNA with no constraints.

Before answering question 2, we first note that the conditional probability is 45.32*%* that a 52-nt RNA codes in both reading frames 0,1 assuming that it begins by the slippery heptamer UUUUUUA is 23.14*%*, and that the conditional probability that a 52-nt RNA codes in reading frame 1, given that it begins by the slippery heptamer UUUUUUA *and* that it already codes in reading frame 0 45.32*%* – i.e. *P*(*A*|*B*,*C*)=0.4532, where event *A* is that a 52-nt RNA codes in reading frame 0, event *B* is that the 52-nt RNA contains slippery heptamer UUUUUUA, and event *C* is that reading frame 0 of the 52-nt RNA contains no stop codon. In contrast, the conditional probability that a 52-nt RNA codes in reading frame 0 assuming that it begins by the slippery heptamer UUUUUUA is 51.06*%*.

Indeed, using RNAsampleCDS, we determine that the number *x*_1_ of 52-nt RNAs beginning by UUUUUUA and which code in both reading frames 0,1 is 2.86451·10^26^. In contrast, the number *x*_2_ of 52-nt RNAs beginning by UUUUUUA and which code in reading frame 0 is *x*_2_=16·61^14^·4=6.32117·10^26^, since there are 16 codons that begin by A, a choice of 61 coding codons for the remaining 14 residues (since the first two residues must be FF and the third residue have a codon beginning by A), times 4 for the last nucleotide to ensure the RNA length is 52. The number *x*_3_ of all 52-nt RNAs that begin by UUUUUUA is clearly 4^45^=1.23794·10^27^. These computations justify the previous probabilities, and suggest the potential utility of RNAsampleCDS when speculating about molecular evolution.

To answer question 2, we used RNAsampleCDS to generate 200,000 52-nt RNA sequences, each of which contains the slippery sequence UUUUUUA and codes 17-mer peptides in both reading frames 0 and 1. Executing RNAshapes as previously described yielded an average probability of stem-loop formation of 59.8*%* with standard deviation of 36.7*%*, and average stem-loop MFE of −8.06 kcal/mol with standard deviation 3.58 kcal/mol.

To answer question 3, we extracted 145 52-nt Pol-Gag overlapping FSS sequences in family RF00480 from the Rfam 12.0, of which 133 sequences remained after disambiguation and removal of sequences containing gaps or stop codons. For each of the 133 sequences, we generated 100,000 sequences using RNAsampleCDS, each of which begins by the same initial 7 nucleotides of the Rfam sequence constituting a slippery sequence (since most but not all RF00480 sequences begin with UUUUUUA), and which code peptides *p* [resp. *q*] having BLOSUM62 similarity of at least +1 with the corresponding amino acids of the 17-mer peptide coded by the Rfam sequence in frame 0 [resp. 1].

**Fig. 5:**
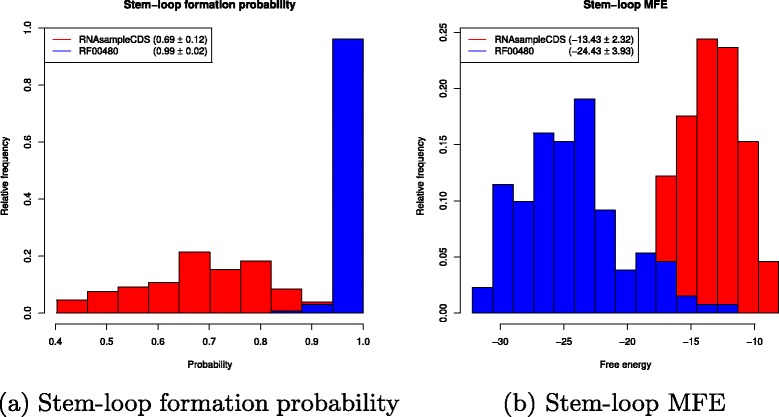
For each of 131 52 nt frameshift stimulating signals (FSS) from family RF00480 from the Rfam 12.0, RNAsampleCDS generated 100,000 RNAs that have the same slippery sequence as the Rfam sequence, and code 17-mer peptides *p* [resp. *q*] in reading frame 0 [resp. 1] each of whose amino acids has BLOSUM62 similarity of at least +1 with the corresponding amino acid in the Pol [resp. Gag] peptide coded by the Rfam sequence. Stem-loop formation probability, *P*(**[** **]**), and stem-loop minimum free energy (MFE) were computed by RNAshapes [21] with the command RNAshapes -q -m ‘[]’. **a** Average stem-loop formation probability for 100,000 sequences sampled from RNAsampleCDS for each RF00480 sequence (red); stem-loop formation probability of HIV-1 frameshift stimulating signals from RF00480 (*blue*). Overall mean RNAsampleCDS samples is 69*%*±12 (*red*), while that for the RF00480 sequences is 99.3±2.2 (*blue*). **b** Average stem-loop MFE for 100,000 sequences sampled by RNAsampleCDS for each RF00480 sequence (red); stem-loop minimum free energy for HIV-1 frameshift stimulating signals from RF00480 (*blue*). Overall mean for RNAsampleCDS samples is 13.43±2.32 kcal/mol (*red*), while that for RF00480 sequences is −24.43±3.91 kcal/mol (*blue*)

After removing two outliers (discussed shortly), we have the following statistics for the remaining 131 sequences from RF00480. Average probability of stem-loop formation for RF00480 is 99.3±2.2*%*, and average stem-loop MFE is −24.43±3.91 kcal/mol. For the collection of 100,000 sequences generated by RNAsampleCDS for each sequence from Rfam family RF00480, coding BLOSUM62 +1 similar peptides to those coded by the Rfam sequence, the average stem-loop formation probability is is 69±12*%*, and average stem-loop MFE is −13.43±2.32 kcal/mol. Figure 5 a and b depict respectively the stem-loop formation probabilities and stem-loop minimum free energies. In contrast, a similar computational experiment using RNAsampleCDS shows that the average probability of stem-loop formation is 98.1*%*±8.1 if each sampled sequence is required to code *exactly* the same peptides as those from HIV-1 in RF00480. This answers question 4.

The previous analysis was performed for 131 Rfam sequences, obtained after removal of the sequences AF442567.1/1455-1506 and L11798.1/1290-1341, from the set of 133 Rfam sequences obtained from 145 sequences in RF00480, after disambiguation and removal of sequences containing gaps or stop codons. These two sequence were removed as outliers, since their stem-loop formation probabilities were respectively 53.1*%* and 55.5*%* – far removed from the average of 99.3±2.2*%* of the remaining sequences. GenBank annotations indicate that AF442567.1 is highly G to A hypermutated with very many, mostly in-frame, stop codons throughout the genome, and that the Gag gene of L11798.1 has a premature termination at position residue 46.

**Fig. 6:**
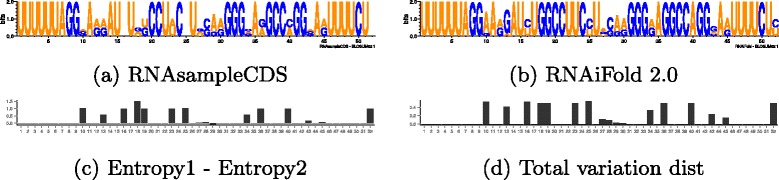
**a** Sequence logo from RNAsampleCDS for *all* 8,819,712 sequences that code peptides *p* [resp. *q*], each of whose amino acids has BLOSUM62 similarity ≥+1 with the corresponding amino acids of the Pol [resp. Gag] 17-mer peptides FFREDLAFPQGKAREFS [resp. FLGKIWPSHKGRPGNFL] in AF033819.3/1631-1682. The PSSM is (exactly) computed by RNAsampleCDS with flag -pssm, and the logo plot was produced using WebLogo [26]. The average pairwise Hamming distance is 10.92±4.32 (length-normalized value of 0.21±0.083), when computed with a random sample of 1000, 5000, and 10,000. **b** Sequence logo for all 1196 sequences determined by RNAiFold 2.0 to fold into the frameshift stimulating signal (FSS) given by the MFE structure from AF033819.3/1629-1682 and code peptides P,Q, each of whose BLOSUM62 similarity with the Gag,Pol peptides in the overlap region is greater than or equal to +1. The average pairwise Hamming distance is 5.80±1.84 (length-normalized value of 0.11±0.035). **c** The position-dependent entropy is defined by *H*_*i*_=−*p*_*A*_ ln*p*_*A*_−*p*_*C*_ ln*p*_*C*_−*p*_*G*_ ln*p*_*G*_−*p*_*U*_ ln*p*_*U*_ for each nucleotide position *i*=1,…,52. Subfigure (**c**) shows the position-dependent *difference* ${H^{a}_{i}} - {H^{b}_{i}}$ in entropies of (**a**) minus (**b**). **d** Position-dependent total variation distance $\delta (\pi _{1,i},\pi _{2,i}) = 1/2 \cdot \sum _{x \in \{A,C,G,U\}} |\pi _{1,i}(x)-\pi _{2,i}(x)|$ in the 52 nt region of the Gag-Pol overlap in the HIV-1 genome (GenBank AF033819.3/1631-1682) that contains the frameshift stimulating signal (FSS). Here *π*_1,*i*_ resp. *π*_2,*i*_ is the mononucleotide frequency at position *i* of the PSSM in the left resp. right panel. If total variation distance is zero, then it is suggestive that the coding constraint automatically may already entail the FSS secondary structure constraint

Together, these results show that stem-loop formation is a consequence of the *precise* HIV-1 Gag and Pol 17-mer peptides, but not of BLOSUM62 +1 similar peptides. As well, stem-loop formation probability is not statistically different (T-test) between random sequences, sequences that have no stop codon in reading frame 0 or 1, and sequences that code peptides having BLOSUM62 similarity of at least +1 to HIV-1 peptides. To determine particular nucleotide positions in the 52-nt FSS that appear to be critical in stem-loop formation, we computed the position-dependent nucleotide frequency (PSSM), denoted by *π*_1_, for 200,000 sequences generated by RNAsampleCDS that begin by the slippery sequence UUUUUUA, and code peptides *p* [resp. *q*], each of whose amino acids has BLOSUM62 similarity greater than or equal to 1 with the corresponding amino acids of the Pol [resp. Gag] 17-mer peptides FFREDLAFPQGKAREFS [resp. FLGKIWPSHKGRPGNFL] coded in AF033819.3/1631-1682. Using RNAiFold 2.0, we also computed the PSSM, denoted by *π*_2_, for all possible sequences that begin by slippery heptamer UUUUUUA, and fold into the MFE structure of AF033819.3/1629-1682 shown in Fig. 1 a, and which code peptides that are BLOSUM62 +1 similar to the peptides coded by AF033819.3/1631-1682. We then computed the position-dependent total variation distance between *π*_1_ and *π*_2_, defined by $\delta (\pi _{1,i},\pi _{2,i}) = 1/2 \cdot \sum _{x \in \{A,C,G,U\}} |\pi _{1,i}(x)-\pi _{2,i}(x)|$, where *π*_1,*i*_ resp. *π*_2,*i*_ denotes the mononucleotide frequency at position *i* of the PSSM for sequences generated by RNAsampleCDS resp. RNAiFold 2.0. With the exception of specific regions, the total variation distance is close to zero, thus pinpointing critical nucleotides necessary for stem-loop formation of the FSS. Figure 6 a, b display the sequence logo for the PSSM *π*_1_ and *π*_2_, and Fig. 6 c and d respectively depict the position-dependent entropy and total variation distance.

To answer question 5, we used RNAiFold 2.0 with target structure as depicted in Fig. 1 a, in order to generate 200,000 52-nt RNA sequences, each containing the slippery sequence UUUUUUA and each folding into the target structure. We determined that 61.91*%* of these sequences have no stop codon in reading frames 0 or 1. The percentage of sequences that have no stop codon in reading frame 0 [resp. 1] alone is somewhat higher, with value 78.7*%* [resp. 79.59*%*]. We additionally determined that the average base pair distance between the MFE structure of the sampled sequences and the target FSS secondary structure is 2.04 and average ensemble defect is 3.58.

The probability of stem-loop formation for frameshift stimulating signal (FSS) regions of HIV-1 is close to 1, with average value of 99*%*±2 for RF00480 as shown in Fig. 5 a. This value is much larger than that of random 52-nt RNAs (≈61*%*), or 52-nt RNA having no stop codons in reading frames 0 or 1 (≈60*%*), or even 52-nt RNA coding peptides in reading frames 0,1 with BLOSUM62 similarity of at least +1 to HIV-1 peptides (≈69*%*). It follows that coding BLOSUM62 +1 similar peptides to those of HIV-1 at most slightly induces stem-loop formation. Yet the probability that stem-loop structures do not have a stop codon in either reading frame 0 or 1 is only about 62%, without requiring that the peptides be similar to those of HIV-1. It follows that BLOSUM62 +1 similarity to HIV-1 peptides cannot induce the required stem-loop FSS structure, nor can the target FSS structure from Fig. 1 a induce BLOSUM62 +1 similarity to HIV-1 peptides. We speculate that starting from a genomic region that codes a polyprotein similar to that of Gag, a series of pointwise mutations could slowly induce a stem-loop FSS structure and at the same time slowly create a Pol-like reading frame. Although speculative, it is possible to create an adaptive walk or Monte Carlo program to test the likelihood of this hypothesis, using intermediate sequences generated by RNAsampleCDS and RNAiFold2.0.

As shown in Fig. 6 a, the average pairwise Hamming distance of sequences generated by RNAsampleCDS with the overlapping coding constraint and the slippery sequence constraint is 10.92±4.32 (length-normalized value of 0.21±0.083), when computed with a random sample of 1000, 5000, and 10,000. As shown in Fig. 6 b, the average pairwise Hamming distance of sequences generated by RNAiFold with the frameshift stimulating structure (FSS) constraint, overlapping coding constraint and the slippery sequence constraint is 5.80±1.84 (length-normalized value of 0.11±0.035). Essentially, this means that approximately 11% of the positions (pairwise) are different for RNAiFold sampled sequences, compared with approximately 21% of the positions (pairwise) for RNAsampleCDS, compared with 81% of the positions (pairwise) for random RNA in positions 8-52 (i.e. after the fixed 7 nt slippery sequence). The greatest reduction in pairwise Hamming distance appears to be due to overlapping coding constraints, with an additional small reduction due to the FSS structural constraint.

### HCV programmed −1 and + 1 frameshifts

**Fig. 7:**
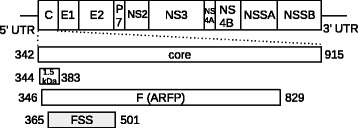
Organization of the initially triple, then double overlapping reading frame region of hepatitis C virus (HCV) (GenBank M62321.1). The top gene organization map is adapted from Fig. 1 a of [23]. All coding regions mentioned in the following include a terminal stop codon. The second line depicts the core in-frame protein, coded in nucleotides 342–915. Next, a 1.5 kDa protein is coded in nucleotides 344–383, while protein F is coded in nucleotides 346–829. The double stem-loop frameshift stimulating signal (FSS) is found at nucleotides 365-501; the FSS structure is depicted in Fig. 8

**Fig. 8:**
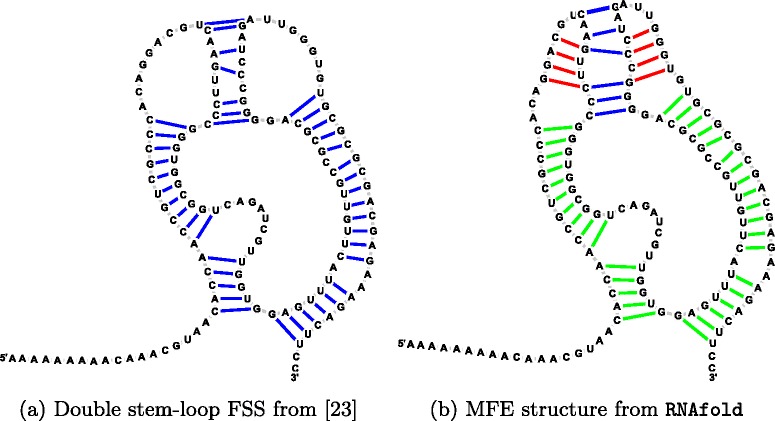
HCV ribosomal frameshift stimulating signal (FSS). **a** Proposed pseudoknotted structure from [23]. **b** Minimum free eneergy (MFE) structure computed by RNAfold 2.1.9 (*green*, *red*), with added pseudoknot (*blue*). Green arcs indicate common base pairs; *red arcs* indicate base pairs predicted by RNAfold but not present in the structure from [23]; *blue arcs* indicate pseudoknot base pairs from the model proposed by [23] that are absent from the RNAfold MFE structure. Figures produced using jViz [27]

There is both in vitro and in vivo experimental evidence for a -2/+1 (hereafter designated as +1) and -1/+2 (hereafter designated as +2) programmed ribosomal frameshift in the core protein of the hepatitis C virus (HCV) [23]. The +1 frameshift produces a 17 kDa protein called protein F (Frameshift), also designated as ARFP (Alternative Reading Frame Protein). In addition, the +2 frameshift produces a 1.5 kDa protein. As measured by in vitro assays, the +1 ribosomal frameshift efficiency is ∼12−15*%*, while the +2 ribosoma frameshift efficiency is ∼30−45*%* [23]. Figure 7 depicts the organization of the overlapping coding region for the HCV genome (GenBank M62321.1), including a double stem-loop RNA structure designated as *frameshift stimulating signal* (FSS) depicted in Fig. 8. According to [23], the frameshift is caused by a poly-A slippery sequence (A AAA AAA AAC) in the triple coding region, although a mutated slippery sequence (A AGA AAA ACC) has also been shown to cause a frameshift, but with a lower efficiency.

**Fig. 9:**
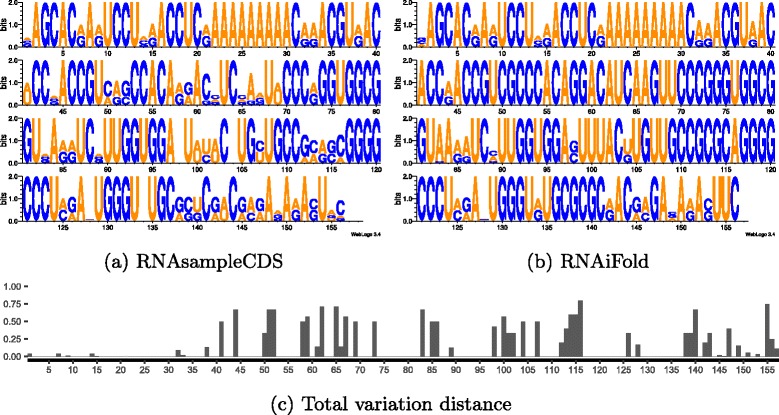
**a** Exact sequence logo determined by RNAsampleCDS for all 2.55×10^17^ sequences, whose initial 39 nucleotides code amino acids having BLOSUM62 +1 similarity to the corresponding amino acids from each of the three reading frames in the triple overlapping coding region 344-383 of the reference HCV genome, and whose remaining nucleotides code amino acids having BLOSUM62 +1 similarity to the corresponding amino acids from each of the two reading frames in the double overlapping coding region 383-501 of the reference HCV genome. **b** Sequence logo determined by RNAiFold 2.0 for the more than 11 million sequences that fold into the HCV FSS structure depicted in Fig. 8, whose initial 39 nucleotides code BLOSUM62 +1 amino acids having BLOSUM62 +1 similarity to the corresponding amino acids from each of the three reading frames in the triple overlapping coding region 344-383 of the reference HCV genome, and whose remaining nucleotides code amino acids having BLOSUM62 +1 similarity to the corresponding amino acids from each of the two reading frames in the double overlapping coding region 383-501 of the reference HCV genome. **c** Total variation distance shown for each nucleotide position, determined by computing the total variation distance between the position-specific profiles of (**a**) and (**b**)

Out of 6,589 sequence hits for the HCV1 frameshift signal for the LANL HCV database (www.hcv.lanl.gov), we found that 94*%* of the sequences started with (A AGA AAA ACC). Furthermore, downstream of the slippery sequence a double stem-loop structure facilitates translational frameshifting (Fig. 8). For this analysis, we took nucleotides 344-500 from the 9401 nt HCV subtype 1a genome (GenBank M62321.1)[23], corresponding to the region starting at the triple coding region and extending to the end of double-stem loop. Using RNAsampleCDS we computed the logo plot for all sequences that code BLOSUM62 +1 similar peptides to those coded by the reference genome (Fig. 9 a). Using RNAiFold 2.0[24], we generated more than 11 million sequences that fold into the double-stem loop structure indicated in Fig. 8 and which have BLOSUM62 similarity of at least +1 to the reference genome peptides (Fig. 9 b). Although RNAiFold 2.0 does not support pseudoknot structures, by providing structural compatibility constraints, we ensured that every sequence returned by RNAiFold 2.0 has the property that the nucleotides, which participate in the “kissing hairpin” model of Fig. 1 a of [23], can indeed form a base pair together. Note that the set of all sequences returned by RNAiFold 2.0, which satisfy both the coding and structural requirements, forms a proper subset of the set of all sequences returned by RNAsampleCDS, which are required to satisfy only the coding requirements. Figure 9 c depicts the total variation distance between these sequence two profiles. At positions where the total variation distance is zero, the secondary structure is likely to be *induced* by the overlapping coding constraints. Indeed, a mutation in such positions could lead to a disruption of the double stem-loop or to a modification of the amino acid in one of the overlapping reading frames. Our results from Fig. 9 c agree with experimental evidence showing that modifications of nucleotides at positions 64, 91, 130 and 137 lead to *detrimental mutations* for the hepatitis C virus [25]. Mutations at these positions resulted in an attenuated HCV infection in chimpanzee. According to our analysis, an introduction of mutations at positions whose variation distance is much greater than zero, should allow the disruption of the double-stem loop with minimal effects on the protein function. This hypothesis could be tested experimentally. 

To further investigate whether the overlapping coding requirement of HCV possibly induces the FSS double stem-loop structure, we proceeded in a manner analogous to that for our HIV-1 analysis. We sampled 100,000 RNA sequences using RNAsampleCDS with BLOSUM62 similarity of +1 and 0 to the reference peptides in each reading frame. Using RNAshapes, we computed the average Boltzmann probability of formation of a double-stem loop with shape **[ ]** **[ ]**, in the sampled RNA sequences as well as 6,589 sequences from LANL database (Additional file 1: Figure S5). Average Boltzmann probability of the double stem-loop shape **[ ]** **[ ]** is 19% [resp. 9%] for BLOSUM62 similarity of +1 [resp. 0], compared with 98% probability for the sequences from LANL HCV database. In contrast, dinucleotide shuffles of sequences generated by RNAsampleCDS having BLOSUM62 +1 similarity to the reference peptides have average probability of 5% of double stem-loop formation, while the probability double stem-loop formation is 6% for random RNA sequences generated with probability of $\frac {1}{4}$ for each nucleotide. Additional file 1: Figure S5 displays average double stem-loop probability and free energy results for the HCV overlapping coding region, which are analogous to results for HIV-1 presented in Fig. 5.

## Conclusion

In this paper, we have developed the novel program RNAsampleCDS, the only existent program which computes the number of RNA sequences that code user-specified peptides in one to six overlapping reading frames, as depicted in Fig. 1 b. More importantly, RNAsampleCDS can compute (exact) PSSMs and sample, in an unweighted or weighted fashion, a user-specified number of RNA sequences that code the specified proteins (or code proteins having BLOSUM/PAM similarity that exceeds a user-specified threshold to the given proteins). With extensions to RNAiFold2.0 made in this paper, RNAsampleCDS and RNAiFold2.0 complement each other and together allow one to analyze the HIV-1 Gag-Pol overlapping reading frame and the HCV triple overlapping reading frame in a manner that cannot be supported by any other software, thus augmenting the software arsenal available to evolutionary biologists.

## Additional file

Additional file 1 Supplementary information for “New tools to analyze overlapping coding regions”. (PDF 416 kb)

## Abbreviations

ARFP: Alternative reading frame protein
CPI: Codon preference index
DP: Dynamic programming
FSS: Frameshift stimulating signal
HCV: Hepatitis C virus
HIV: Human immunodeficiency virus
MFE: Minimum free energy
PSSM: Position-specific scoring matrix, also called profile
