# Supplementary material for: New tools to analyze overlapping coding regions
Source: BMC Bioinformatics. 2016 Dec 13;17:530. doi: 10.1186/s12859-016-1389-7 (PMC5155393; doi:10.1186/s12859-016-1389-7)
Supplement: Additional file 1 — Supplementary information for “New tools to analyze overlapping coding regions”. (PDF 416 kb) [file 12859_2016_1389_MOESM1_ESM.pdf]

SUPPLEMENTARY INFORMATION FOR "NEW TOOLS TO ANALYZE OVERLAPPING CODING REGIONS"

A.H. BAYEGAN, J.A. GARCIA-MARTIN, PETER CLOTE
BIOLOGY DEPARTMENT, BOSTON COLLEGE

In this supplement, we discuss the following topics in greater detail: (1) How to use the software `RNAiFold 2.0` [2] to investigate a secondary structure frameshift stimulating signal within an overlapping coding region, as in the case of the HIV-1 Gag-Pol overlap region and the HCV triple overlapping coding region. (2) Computation of the *codon preference index* (CPI), using different control data sets. (3) Analysis of the HIV-1 Gag-Pol synonymous substitution rate with respect to different reading frames. (4) Run time analysis of `RNAsampleCDS`.

1. `RNAiFold 2.0`

In this section, we provide examples for the syntax used to run `RNAiFold 2.0` [2], in order to generate RNA sequences that fold into a user-specified target secondary structure and which code user-specified peptides in possibly overlapping coding regions. For more details, please consult [2]. The command file in Example 1 below generates all RNAs that code the 17-mer peptide FFREDLAFLQGKAREFS [resp. FLGKIWPSYKGRPGNFL] in the Pol [resp. Gag] reading frame 0 [resp. 1]. Although not shown here, `RNAiFold 2.0` allows one to stipulate simultaneous coding requirements in reading frames 0,+1,+2 (but not -0,-1,-2).

```
1. > Example 1: overlapping amino acid constraints; NO secstr
constraint
2. ,,,,,,,,,,,,,,,,,,,,,,,,,,,,,,,,,,,,,,,,,,,,,,,,,,,,
3. NNNNNNNNNNNNNNNNNNNNNNNNNNNNNNNNNNNNNNNNNNNNNNNNNNNN
4. #AAseqcon
5. FFREDLAFLQGKAREFS,FLGKIWPSYKGRPGNFL
6. #AAstartPos
7. 1,2
8. #AAsimilCstr
9. 1
10. #MAXsol
11. 0
```

Line 1 is a comment, while lines 2,3 indicate structure resp. sequence constaints, neither of which apply in the current Example 1. Indeed, a comma in a given position indicates that the nucleotide may be either paired or unpaired (no constraint), and 'N' in a given position indicates any RNA nucleotide can be listed. Lines 4-7 indicate that coding constraint is imposed in positions 1 and 2 (i.e. reading frames 0 and 1), and that the translated peptides are FFKKNGAGSCDPAPDFF resp. FLKKTEPGRVTRLRTFL. Note that '#' is not a comment, but rather part of a 'hashtag' the precedes the value of a parameter. Lines 8,9

Corresponding author: `clote@bc.edu`.

indicate that all returned RNA sequences must code peptides p [resp. q] in reading frame 0 [resp. 1], whose amino acids have BLOSUM62 similarity is at least 1 with the corresponding amino acids of the peptides FFREDLAFLQGKAREFS [resp. FLGKIWPSYKGRPGNFL], in the Pol [resp. Gag] reading frame, as shown in line 5. Lines 10,11 indicate that there is no upper bound on the number of solutions, hence all 6,614,784 solutions are to be generated. See [2] for more detailed explanation of the syntax for `RNAiFold 2.0` command files.

If the parameter for #`AAsimilCstr` had been 0, instead of 1, then `RNAiFold 2.0` could *in theory* generate all 55,552,444,416 solutions – this is due to the fact that memory usage does not depend on the number of solutions in the search space. Examples 3 and 4 show that `RNAiFold 2.0` can generate enormous numbers of sequences, whose MFE structure is identical to a given target structure, and which satisfy possible additional constraints.

Executing `RNAiFold 2.0` on the command file from Example 1 returns all 6,614,784 solutions in user+sys time 3.27 hours (11766.06 sec). In contrast, within 12.96 min (777.87 sec), `RNAsampleCDS` sampled 10 million sequences that code peptides p [resp. q] in the Pol [resp. Gag] reading frame 0 [resp. 1], each of whose amino acids has BLOSUM62 similarity of at least +1 with those of the Pol peptide FFREDLAFLQGKAREFS [resp. Gag peptide FLGKIWPSYKGRPGNFL]. After sorting and removing duplicates, which required an additional 43.0 sec, we obtained 4,758,267 unique solutions. Clearly, `RNAsampleCDS` is much faster tool to sample sequences that code in overlapping reading frames, provided that the sequences returned are not required to fold into a specified target structure, and provided that *all* solutions need not be returned; otherwise `RNAiFold 2.0` is the appropriate tool to apply.

The following `RNAiFold 2.0` command file generates all frameshift stimulating signals, that include the `UUU UUU A` slippery sequence in the Pol reading frame, have (exactly) the minimum free energy structure corresponding to that of GenBank AF033819.3/1631-1681, and code peptides in reading frames 0,1 that have at least BLOSUM62 similarity of +1 with corresponding peptides translated in AF033819.3/1631-1681. There are exactly 1196 solutions for BLOSUM62 threshold of +1, 42,534 solutions for threshold 0, and more than 230,261,152 solutions for threshold -1.

```
1. > Example 2: overlapping amino acid (AA) constraints with target
sec str
2. ......((((((..((((((((((((....))))))))))))...)))))).
3. UUUUUUANNNNNNNNNNNNNNNNNNNNNNNNNNNNNNNNNNNNNNNNNNNN
4. #AAseqcon
5. FFREDLAFLQGKAREFS,FLGKIWPSYKGRPGNFL
6. #AAstartPos
7. 1,2
8. #MAXsol
9. 0
10. #AAsimilCstr
11. 1
```

Since the publication of [2], `RNAiFold 2.0` allows the desired peptides to be entered using PROSITE pattern syntax, given in Example 3 below. The PROSITE patterns below for Pol and Gag peptides were obtained by analyzing those 665 sequences from LANL HIV-1 database which contain slippery sequence UUUUUUA and whose MFE FSS structure is identical to that of Figure 1a of the main paper, the most common structure found in

LANL. The wild card '*' (representing a stop codon) can be used in `RNAiFold 2.0` as shown in the following command file.

```
1. > Example 3: overlapping AA constraints, FSS, PROSITE pattern
syntax
2. ......((((((..((((((((((((....))))))))))))...)))))).
3. UUUUUUANNNNNNNNNNNNNNNNNNNNNNNNNNNNNNNNNNNNNNNNNNNNN
4. #AAseqcon
5.  [F][F][KR][KE][ASTNKHDIG][FWMPRELV][ATPV][SAFCL][HSQPL]\
[SKEHQCRGL][QWDRGE][AQRKGE][SATRKPE][ASWRGEV][FNRKGE]\
[SFPKEL][FSP*YHCL],\
[F][L][KG][NRK][AFNKPVMIL][WCRGL][SHQPL][AFSCPV]\
[STNKPYHQCRL][ASQTNKRG][TDRKGE][SPRG][QRPG][ANP*GE]\
[FNIG][SFNPL][ASFQCIPLV]
6. #AAstartPos
7. 1,2
8. #MAXsol
9. 0
```

Line 5 is a *single* line, where continuation is indicated by a backslash (shown as displayed in order to fit column dimensions). Notice as well the presence of the comma in line 5, which separates the amino acid ccoding constraints for reading frame 0 from those for reading frame 1. We could have stipulated amino acid constraints in three reading frames by replacing line 5 by three PROSITE patterns separated by commas, and by replacing line 7 by '1,2,3'. Running `RNAiFold 2.0` in the background with the command file of Example 3 for a few weeks, we obtained more than 273,926,421 solutions before we chose to terminate the computation.

The following example returns sequences containing the slippery heptamer UUUUUUA, that fold into the most common secondary structure found in the LANL HIV database and having GC-content between 20-40%. Note that no amino acid constraints are given.

```
1. > Example 4: FSS
2. ......((((((..((((((((((((....))))))))))))...)))))).
3. UUUUUUANNNNNNNNNNNNNNNNNNNNNNNNNNNNNNNNNNNNNNNNNNNNN
4. #minGCcont
5. 20
6. #maxGCcont
7. 40
8. #MAXsol
9. 0
```

By adding two additional lines, respectively containing '#LNS' and '1', `RNAiFold 2.0` applies Large Neighborhood Search (LNS) instead of default Constraint Programming (CP) – see [1] for explanation. In contrast to CP, LNS may perform restarts, leading to multiple occurrences of the same solution. LNS can be faster than CP, especially when the target structure is large; when using LNS for Example 3, we obtained 559,877,906 solutions, of which 440,389,701 were unique, before terminating execution. By running `RNAiFold 2.0` on this command file, along with two other command files where the values 20,40 in lines 5,7 are replaced by 40,60 and by 60,80, a total of 713,134,134 solutions were obtained before we decided to terminate the computations. These examples show that `RNAiFold 2.0` can efficiently generate a very large number of sequences, all of which are guaranteed to fold into the target structure and comply with any additional constraints that may be imposed.

2. Codon usage analysis

We begin by recalling the following definitions. For RNA sequence $\mathbf{a} = a_0, \ldots, a_{3n}$ which codes n-mer peptides in reading frames $0, 1$, for codon $w \in (\{A, C, G, U\})^3$ and reading frame $r \in \{0, 1\}$, define $f_{(w,\mathbf{a},r)}$ to be the number of occurrences of codon w in reading frame r of $\mathbf{a}$, and for amino acid AA, define $f_{(AA,\mathbf{a},r)}$ to be the number of occurrences of codons coding AA in reading frame r of $\mathbf{a}$. Define the *observed codon preference* in reading frame r in sequence $\mathbf{a}$ by $p_{obs}(w, \mathbf{a}) = \sum_{r=0}^{1} f_{(w,\mathbf{a},r)} / \sum_{r=0}^{1} f_{(AA,\mathbf{a},r)}$. If S is a set of mRNAs of length $3n + 1$, each of which codes n-mer peptides in both reading frames $0, 1$, then define the *observed codon preference* in S by $p_{obs}(w, S) = \sum_{r=0}^{1} \sum_{\mathbf{a} \in S} f_{(w,\mathbf{a},r)} / \sum_{r=0}^{1} \sum_{\mathbf{a} \in S} f_{(AA,\mathbf{a},r)}$. Define the *codon preference index* $I(w)$ of codon w in S by $I(w) = p_{obs}(w, S)/p_{obs}(w, S')$, where S' is a *control* set of mRNAs of length $3n+1$. In some cases below, we consider only one reading frame, as when analyzing the Gag only and Pol only reading frames, in which case we define the observed codon preference with respect to the appropriate reading frame r alone: $p_{obs}(w, \mathbf{a}) = f_{(w,\mathbf{a},r)}/f_{(AA,\mathbf{a},r)}$ and $p_{obs}(w, S) = \sum_{\mathbf{a} \in S} f_{(w,\mathbf{a},r)} / \sum_{\mathbf{a} \in S} f_{(AA,\mathbf{a},r)}$. By definition, codon preference index $I(w)$ values less than 1 (greater than 1) indicate that codon w is avoided (preferred).

In the following, we consider the *general* formulation of the forward and backward partition function, defined to account for all six reading frames $+0, +1, +2, -0, -1, -2$. As mentioned in the paper, this requires the consideration of 5-tuples $s = s_0 s_1 s_2 s_3 s_4$. For simplicity of exposition in the main paper, the forward and backward partition function were defined only for reading frames 0 and 1, for which reason, we considered 4-tuples $s = s_0 s_1 s_2 s_3$. In the sequel, $p_{obs}(w, S)$ can be calculated by counting codons in S, and $p_{obs}(w, S)$ is computed utilizing the forward and backward partition functions as follows:

$$\begin{aligned} f_{(w,\mathbf{a},r)} &= \sum_{k=1}^{n} \sum_{s \in L_k} I[w \in \text{reading frame } r \text{ of } \mathbf{a}] \cdot ZF(k-1, s_0, s_1) \cdot ZB(k, s_3, s_4) \\ f_{(AA,\mathbf{a},r)} &= \sum_{w \in \text{all codons translating AA}} f_{(w,\mathbf{a},r)} \end{aligned}$$

where $ZF[0, ch1, ch2] = 1$ and $ZB[n, ch1, ch2] = 1$ for $ch1, ch2 \in \{A, C, G, U\}$.
Similarly, $p_{obs}(w, S', gc)$ can be defined as the probability of observing w in sequences of S' with GC-content in range $gc = [gcl, gcu]$:

$$\begin{aligned} f_{(w,\mathbf{a},r,gc)} &= \sum_{k=1}^{n} \sum_{s \in L_k} \sum_{x_1=1}^{3k+2} \sum_{x_2=1}^{3(n-k)+2} I[w \in \text{reading frame } r \text{ of } \mathbf{a} \text{ and gcl} \le x_1 + x_2 + \text{GCcount}(s_2) \le \text{gcu}] \\ &\cdot \; ZF(k-1, x_1, s_0, s_1) \cdot ZB(k, x_2, s_3, s_4) \\ f_{(AA,\mathbf{a},r,gc)} &= \sum_{w \in \text{all codons translating AA}} f_{(w,\mathbf{a},r,gc)} \end{aligned}$$

where $ZF[0, 0, ch1, ch2] = 1$ and $ZB[n, 0, ch1, ch2] = 1$ for $ch1, ch2 \in \{A, C, G, U\}$.

With this notation, Figures S2, S3, and S4 depict heat maps for the codon preference index $I(w)$, computed over 5,125 sequences from the LANL HIV-1 database. Figure S2 shows the heat map of $I(w)$ computed for 5,125 Gag and Pol sequences obtained from the LANL HIV-1 database. Five columns are indicated for each codon:

Column 1: S is the set of Gag sequences from the LANL HIV-1 database without the overlapping region. S' is the collection of $\sim 8 \times 10^{232}$ sequences that code proteins in S in the Gag reading frame.

Column 2: S is the set of Pol sequences from the LANL HIV-1 database without the overlapping region. S' is the collection of sequences that code proteins in S in the Pol reading frame. The number of sequences in S' is so huge that is could not be exactly defined.

Column 3: S is the set of Gag-Pol overlapping sequences from the LANL HIV-1 database. S' is the collection of $1,204,620$ sequences that code proteins in S in both Gag and Pol reading frames.

Column 4: S is the set of Gag-Pol overlapping sequences from the LANL HIV-1 database. S' is the collection of $\sim 10^{34}$ sequences that code proteins in S having BLOSUM62 similarity of at least +1 in both Gag and Pol reading frames.

Column 5: S is the set of Gag-Pol overlapping sequences from the LANL HIV-1 database. S' is the collection of $1,022,784$ sequences that have GC-content of ± 5 to at least one sequence in S and code the same proteins as S in both Gag and Pol reading frames.

The heat map in Figure S3 depicts values of $I(w)$ computed for the same dataset as above. In all columns of Figure S3, S is the set of Gag-Pol overlapping sequences from the LANL HIV-1 database. Note that this is the same S collection used for Figure 3 of the main text. The control set S' (see main text for explanation) in columns 1 and 2 is the collection of sequences that code any protein of length 68 in a single reading frame. However, in columns 3-5, S' is the collection of sequences that code any protein of length 68 in both +0 and +1 reading frames. Mean peptide length in the overlapping region of the dataset is 68. Note that the codon preference index (CPI) computed in Figure S3 is with respect to all possible coding sequences regardless of amino acid coded, and so is natural generalization of the method of [3] to the case of overlapping reading frames.

Figure S4 shows the standard deviation of $I(w)$ for the codons of each amino acid. Here, $I(w)$ is computed as in Figure 4 of the main text. Arginine is the most varied and thus the most optimized amino acid in the Gag-Pol overlapping region.

3. Synonymous substitution rate analysis

To further clarify that Gag-Pol overlapping region is under high evolutionary constraint we used FRESCo, a phylogenetic codon model-based to find regions in excess synonymous constraint to analyse 200 Gag-Pol sequences from LANL HIV-1 database. The phylogenetic tree expected as an input to FRESCo was built by `RAxML v.8` [5]. As Figure S1a indicates, in the starting and ending regions of Pol where it has overlap with Gag and Vif genes, synonymous substitution rate is low. Figure S1b also indicates a sudden drop in the

the synonymous substitution rate for 200 artificial Gag-Pol sequences in which an extra nucleotide 'U' is inserted at the end of Gag to coordinate the reading frames.

4. Run time analysis of `RNAsampleCDS`

With the exception of Algorithm 1, which uses breadth first search (BFS), all algorithms run in linear space and time. For the benefit of readers unfamiliar with algorithmic complexity, we provide a brief discussion of the linear run time, and then use least squares fitting to give an estimate of the run time constant in computational experiments.

In Algorithm 1, our method explicitly constructs a *prefix tree* (also called *trie* in computer science), whose root is the empty string, such that nodes at depth k are 4-nt RNAs $\mathbf{t} = t_0t_1t_2t_3$ with the property that the first nucleotide of $\mathbf{t}$ is identical with the last nucleotide of the parent $\mathbf{s} = s_0s_1s_2s_3$ of $\mathbf{t}$ – and of course, that the merge of all 4-tuples from the root to $\mathbf{t}$ satisfies the (overlapping) coding requirement in both reading frames. It follows that every mRNA that satisfies the coding requirement appears as the merge of a unique path from root to leaf, hence the run time and memory requirements are $O(N)$, where there are N possible solution mRNA sequences.

Given overlapping n-mer peptides, Algorithm 2 uses dynamic programming to compute the forward partition function $ZF(k, ch)$ and the backward partition function $ZB(k, ch)$ for $k = 0, \ldots, n$ and each nucleotide $ch \in \{A, C, G, U\}$. When inductively computing the value of $ZF(k, ch)$ [resp. $ZB(k, ch)$], finitely many arithmetic operations are applied to the previously computed values $ZF(k-1, A)$,ZF(k-1,C), $ZF(k-1, G)$,ZF(k-1,U) [resp. $ZB(k+1, A)$,ZB(k+1,C), $ZB(k+1, G)$,ZB(k+1,U)] are performed. It follows that there are $O(n)$ many inductive steps, each of which requires constant time, hence the run time is $O(n)$, as well as the memory requirements. Similarly, the computation of PSSM (Algorithm 3), of positional codon frequency (Algorithm 4), and both unweighted sampling (Algorithm 5) and weighted sampling (Algorithm 6) require linear time and space. It should be noted that in both sampling algorithms, the run time is $O(n)$ to first compute the forward and backward partition functions ZF, ZB, and then for *each* sequence that is sampled, the run time is $O(n)$. Ultimately, the run time for the sampling algorithms depends on the number N of desired samples, so the overall run time is $O(n + N)$, where n is the length of the peptides that must be coded, and N is the number of samples. An estimate of the actual run time constants for n and N are given next.

The run time for `RNAsampleCDS` is ostensibly linear in RNA sequence length and number of samples to be generated. Using least squares fitting, we can compute the run time as follows. For each sample size N equal to 10^4, 2×10^4, 3×10^4, we generated N samples using `RNAsampleCDS`, which code peptides having $A = 20, 30, 40, \ldots, 160$ many amino acids in all 6 overlapping reading frames (i.e. the only requirement is absence of a stop codon). It follows that sequence length $L = 3 \cdot A + 2$ takes values $62, 92, 122, \ldots, 482$ thus providing 45 data points. Now define M to be the 45×2 matrix, for which $M_{i,1}$ is the sequence length $L \in \{62, 92, \ldots, 482\}$ and $M_{i,2}$ is the number of samples $N \in \{10^4, 2 \times 10^4, 3 \times 10^4\}$ for the ith data point. Define B to be the 45×1 column vector, where B_i is the run time for `RNAsampleCDS` to compute the partition function and generate N samples for the

ith data point. Using the Python function `numpy.linalg.lstsq`, we solved $MX = B$ by least squares to determine that `RNAsampleCDS` computes the partition function in time $\approx 0.58831373 \cdot L$, and samples N RNA sequences of length L in time $\approx 0.00550239 \cdot N$. See Figure S6 for a plot of the run time of `RNAsampleCDS` for this data.

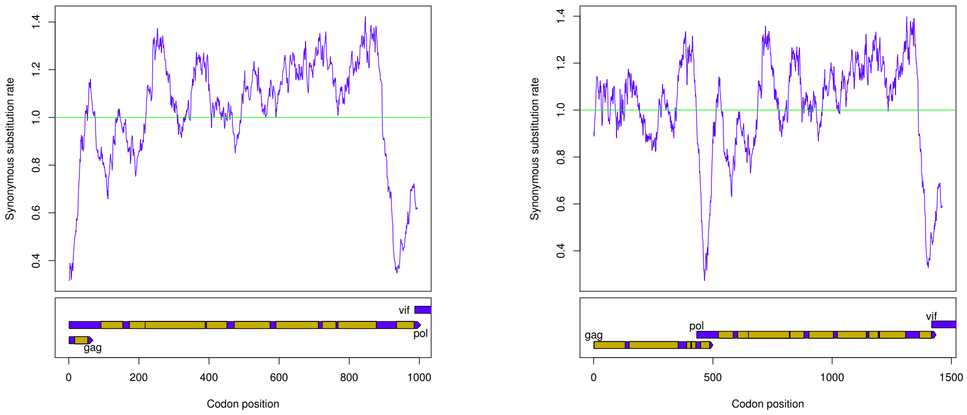

(A) Pol reading frame (B) Artificial

Figure S1. Synonymous substitution rate analysis using `FRESCo` [4], with window size 50 nt, for 200 Pol *(Left)* and 200 modified Gag-Pol *(Right)* sequences from the LANL HIV-1 database. Gag-Pol sequences were modified by inserting one additional nucleotide at the beginning of the overlapping coding region, thus causing the Pol reading frame to be in-frame, rather than -1. Codon positions in the lower panel are based on HXB2 reference sequence. Mature peptides are shown in yellow.

References

[1] J. A. Garcia-Martin, P. Clote, and I. Dotu. RNAiFOLD: a constraint programming algorithm for RNA inverse folding and molecular design. *J. Bioinform. Comput. Biol.*, 11(2):1350001, April 2013.

[2] J. A. Garcia-Martin, I. Dotu, and P. Clote. RNAiFold 2.0: a web server and software to design custom and Rfam-based RNA molecules. *Nucleic. Acids. Res.*, 43(W1):W513–W521, July 2015.

[3] M. Gribskov, J. Devereux, and R. R. Burgess. The codon preference plot: graphic analysis of protein coding sequences and prediction of gene expression. *Nucleic. Acids. Res.*, 12(1):539–549, January 1984.

[4] R. S. Sealfon, M. F. Lin, I. Jungreis, M. Y. Wolf, M. Kellis, and P. C. Sabeti. FRESCo: finding regions of excess synonymous constraint in diverse viruses. *Genome Biol.*, 16:38, 2015.

[5] Alexandros Stamatakis. RAxML-VI-HPC: Maximum likelihood-based phylogenetic analyses with thousands of taxa and mixed models. *Bioinformatics*, 22(21):2688–2690, 2006.

[6] P. Steffen, B. Voss, M. Rehmsmeier, J. Reeder, and R. Giegerich. RNAshapes: an integrated RNA analysis package based on abstract shapes. *Bioinformatics*, 22(4):500–503, 2006.

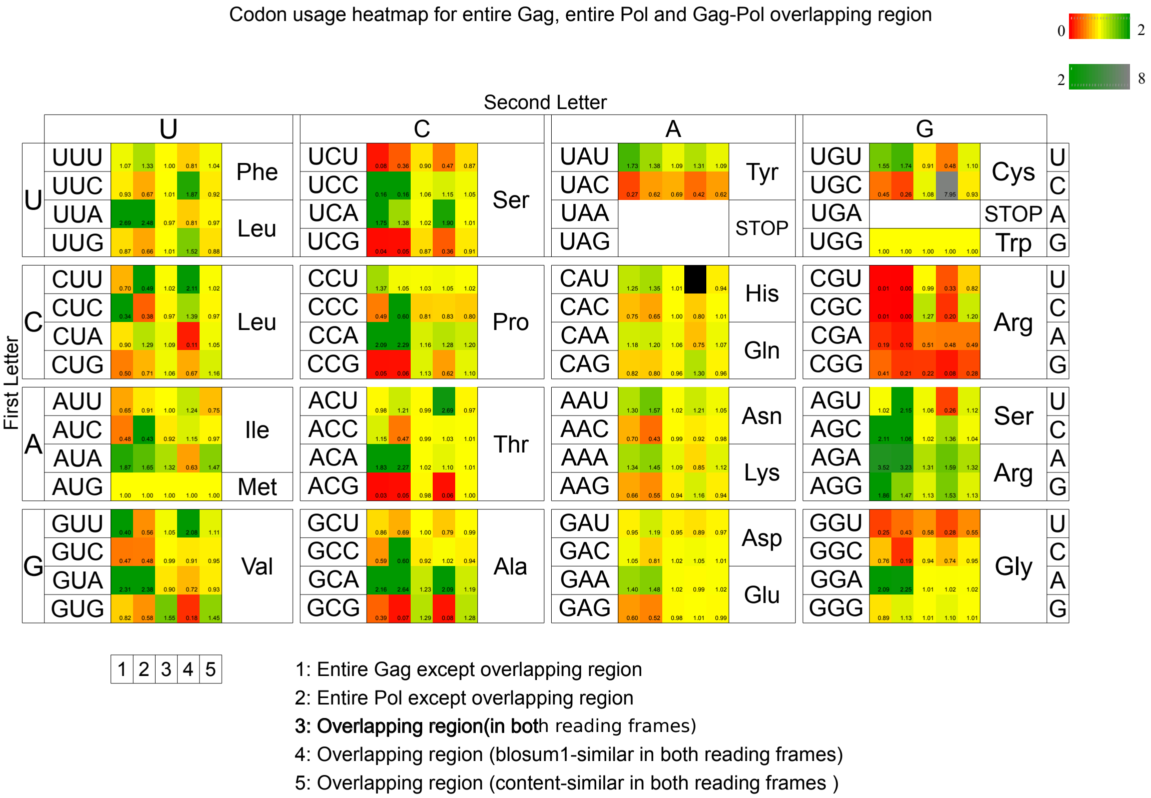

Figure S2. Heat map of the codon preference index (CPI) for a collection of 5,125 Gag, Pol and Gag-Pol overlapping sequences obtained from the LANL HIV-1 database.

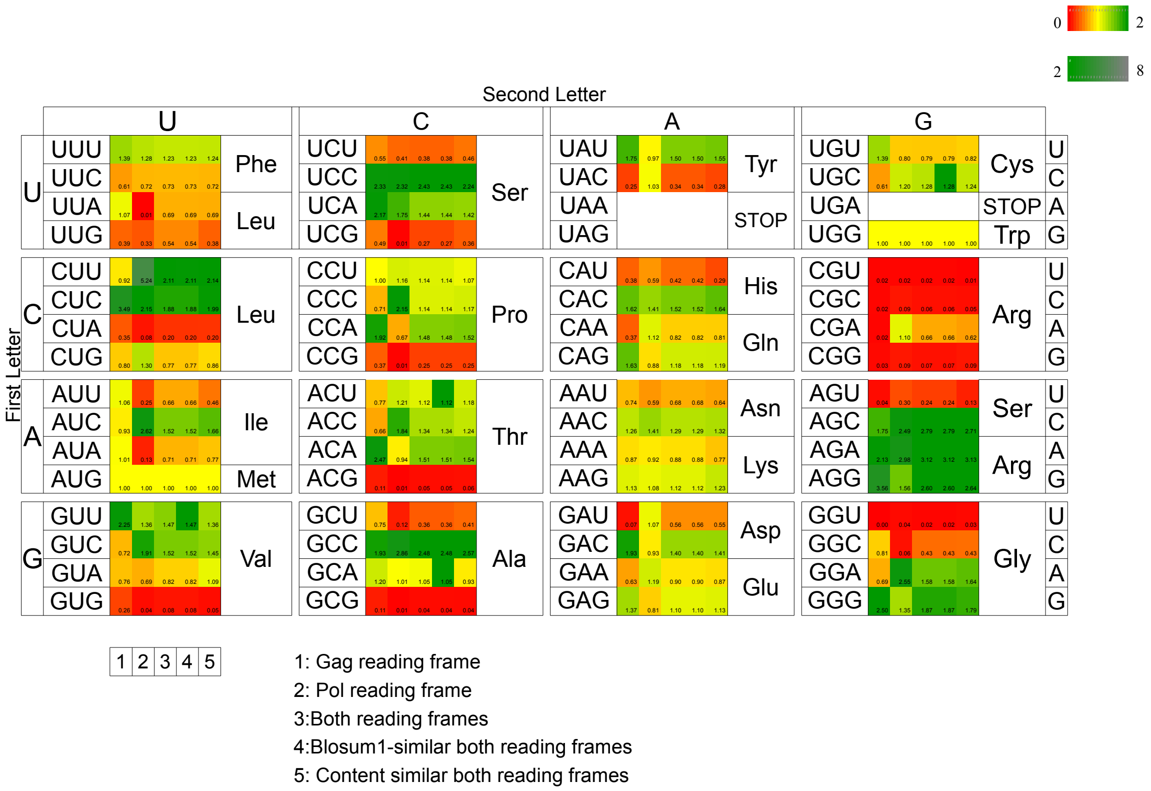

FIGURE S3. Heat map of the codon preference index (CPI) for a collection of 5,125 Gag-Pol overlapping sequences obtained from the LANL HIV-1 database where S' is the collection of sequences coding any amino acid (i.e. not containing a stop codon) in the corresponding reading frames.

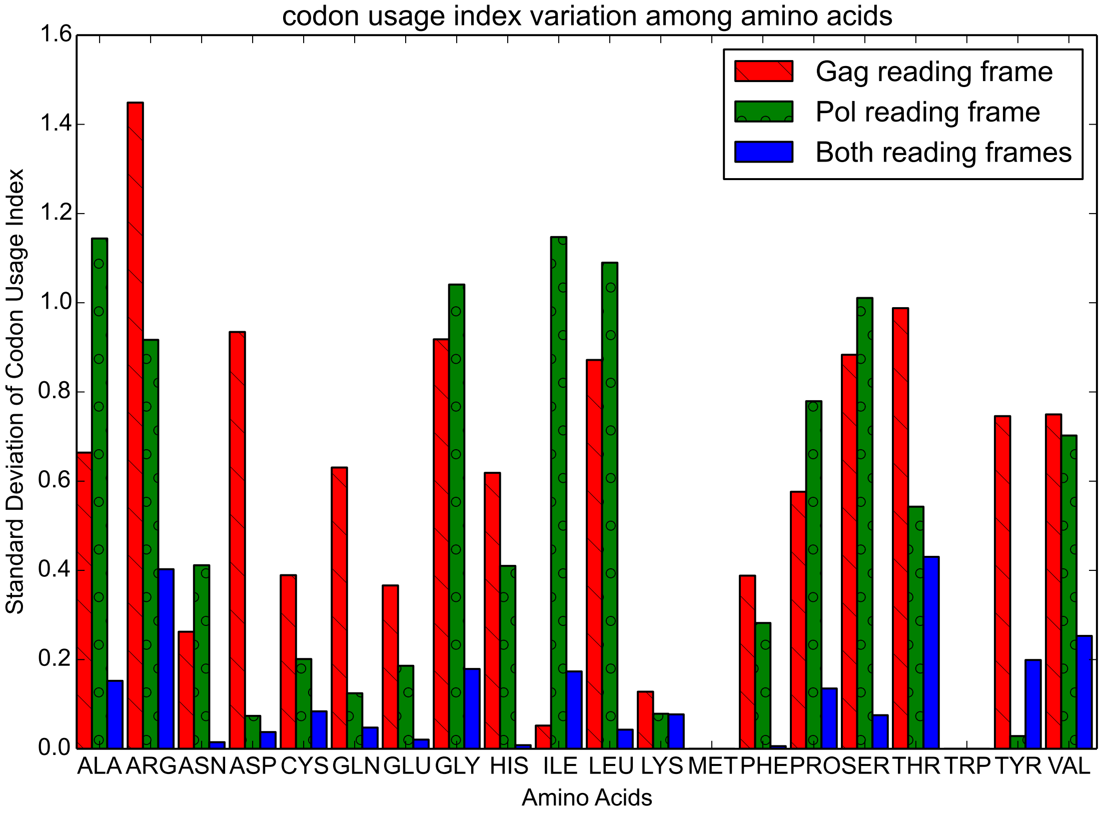

FIGURE S4. Standard deviation of CPI for synonymous codons computed from the Gag-Pol overlapping sequence of 5,125 sequences from the LANL HIV-1 database.

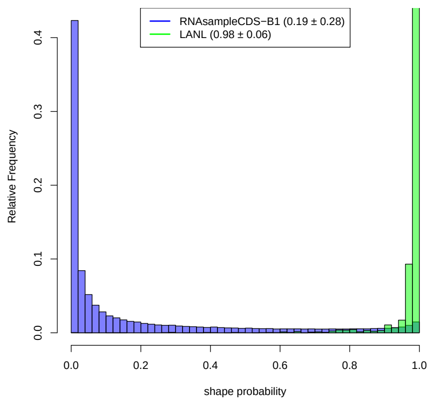

(A) BLOSUM62 +1 vs LANL HCV

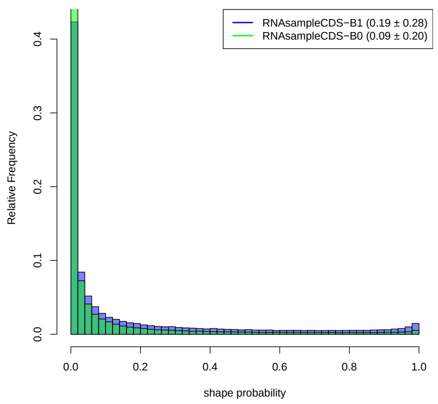

(B) BLOSUM62 +1 vs BLOSUM62 0

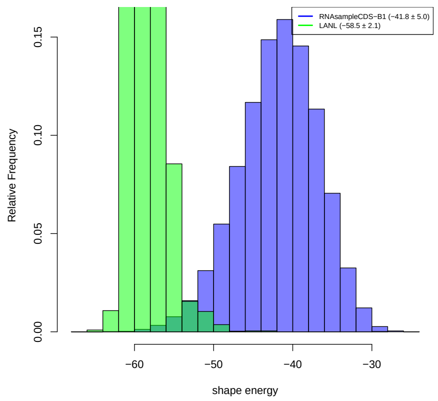

(C) BLOSUM62 +1 vs LANL HCV

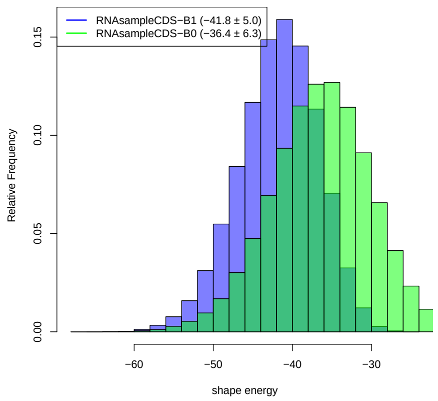

(D) BLOSUM62 +1 vs BLOSUM62 0

FIGURE S5. Using `RNAsampleCDS`, we sampled 100,000 sequences coding peptides having BLOSUM62 +1/0 similarity to the peptides in each overlapping reading frame of the reference HCV1a genome (GenBank M62321.1). Using `RNAshapes` [6], we determined the Boltzmann probability of having a double stem-loop shape `[][]`. We also determined the Boltzmann probability of double stem-loop shape `[][]` in 6,589 sequences from the LANL HCV database. (A) Average double stem-loop probability of BLOSUM62 +1 sequences compared with that of the LANL HCV sequences. (B) Average double stem-loop probability of BLOSUM62 +1 sequences compared with Blosum 0 sequences. (C) Average double stem-loop free energy of BLOSUM62 +1 sequences compared with that of the LANL HCV sequences. (D) Average double stem-loop free energy of BLOSUM62 +1 sequences compared with that of BLOSUM62 0 similar sequences.

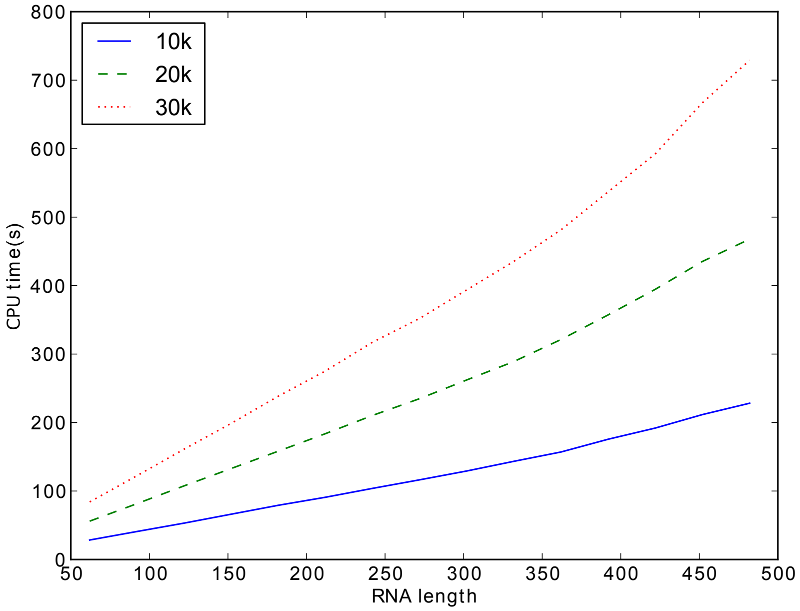

FIGURE S6. Run time for RNAsampleCDS to generate RNA sequences of length L that code peptides in all six reading frames – i.e. a stop codon does not appear in any of the six reading frames. For each sample size N equal to 10^4, 2×10^4, 3×10^4, RNAsampleCDS generated N samples that code peptides having $A = 20, 30, 40, \ldots, 160$ many amino acids. Thus sequence length $L = 3 \cdot A + 2$ takes values $62, 92, 122, \ldots, 482$ thus providing 45 data points. Using least squares fitting, we determine that RNAsampleCDS computes the partition function in time $\approx 0.58831373 \cdot L$, and samples N sequences each of length L in time $\approx 0.00550239 \cdot N$.
